# Supplementary material for: Telomere Maintenance Pathway Activity Analysis Enables Tissue- and Gene-Level Inferences
Source: Front Genet. 2021 Apr 7;12:662464. doi: 10.3389/fgene.2021.662464 (PMC8058386; doi:10.3389/fgene.2021.662464)
Supplement: Supplementary file 1 [file Data_Sheet_1.docx]

Supplementary data

# Improved analysis of telomere maintenance pathway activity allows for tissue and gene-specific inference

Lilit Nersisyan, Arman Simonyan, Hans Binder, Arsen Arakelyan

## Table S1. Functional role of the ALT pathway nodes.

| **Name (alias)** | **Type^a^** | **Description** | **Effect^b^** | **Reference** |
| --- | --- | --- | --- | --- |
| G4 formation; DDR provokation | | | | |
| *G4 quadruplexes are telomeric structures that serve as replication fork barriers and may trigger DNA damage response (DDR) that may in turn recruit ALT factors.* | | | | |
| H3F3A | G | H3.3 histone variant. Suppresses formation of G4 structures at telomeres, which are replication fork barriers at telomeres, trigger fork stalling and restart by homology directed repair, and are HR substrates. The purpose of H3.3 deposition at telomeres remains unclear. | **-** | (Clynes et al., 2015), (Dyer et al., 2017) |
| DAXX-ATRX | C | ATRX/DAXX complex is required for deposition of H3.3 histone variants at telomeres. | **-** | (Dyer et al., 2017) |
| DAXX | G | Member of ATRX/DAXX. Histone chaperone. Binds H3.3 and deposits H3.3-H4 tetramers onto naked DNA. 80% of ALT tumors show mutations in ATRX/DAXX. | **-** | (Dyer et al., 2017), (Lovejoy et al., 2012) |
| ATRX | G | Member of ATRX/DAXX. Chromatin remodelling factor. Binds DAXX to incorporate H3.3 to telomeres. 80% of ALT tumors show mutations in ATRX/DAXX. Re-introduction of ATRX represses ALT. | **-** | (Dyer et al., 2017), (Lovejoy et al., 2012), (Clynes et al., 2015) |
| NuRD-mediated telomere bridge formation (NTB) | | | | |
| *Telomeric bridges are molecular structures that bring together two telomeric strands (chromosomes) as an initial step before strand invasion.* | | | | |
| NR2C2 (TR4) | G | Nuclear receptor. Gets recruited to ALT telomeres. In turn, recruits ZNF827. | **+** | (Conomos et al., 2014) |
| NR2F2 (COUP-TF2) | G | Nuclear receptor. Gets recruited to ALT telomeres. In turn, recruits ZNF827. | **+** |  |
| ZNF827 | G | Zinc finger protein. Is recruited to ALT telomeres via NR2C2 and NR2F2. In turn, recruits NuRD complex. | **+** |  |
| NuRD | C | Chromatin remodeling complex. Is recruited to ALT telomeres by ZNF827. Serves as a bridge between two telomeric strands, and between telomeres and APB. | **+** |  |
| Recruitment of telomeres to APBs (APB) | | | | |
| *Formation of APBs and recruitment of telomeres to APB sites.* | | | | |
| PML | G | Transcription factor. One of the main consituents of APBs. | **+** | (Chung et al., 2012) |
| SMC5/6 | C | A structural maintenance of chromosomes (SMC) complex. NSE2(member)-mediated SUMOylation of shelterin proteins is required for recruitment of telomeres to APBs, as PML has a SUMO-binding pocket. Knock-down of SMC5, SMC6 and NSE2 reduces ALT activity. | **+** | (Aragón, 2018), (Potts and Yu, 2007) |
| SMC5 (RAD18) | G | Member of SMC5/6. | **+** |  |
| SMC6 (Spr18) | G | Member of SMC5/6. | **+** |  |
| NSMCE2 (NSE2) | G | Member of SMC5/6. NSE2-mediated SUMOylation of shelterin proteins is required for recruitment of telomeres to APBs. | **+** |  |
| Strand invastion (SI) | | | | |
| *The release and subsequent hybridization of 3' strand of one telomere to 5' strand of another telomere or telomeric template.* | | | | |
| POT1 | G | Member of shelterin complex, binds ssDNA at telomeres. Antagonizes the binding of RPA to telomeres. | **-** | (Flynn et al., 2012) |
| RPA | I | A non-sequence specific ssDNA binding complex of RPA1, RPA2 and RPA3. Replaces POT1 and recruits ATR to ssDNA at telomeres. | **+** |  |
| RPA1 | G | Member of RPA complex. | **+** |  |
| RPA2 | G | Member of RPA complex. | **+** |  |
| RPA3 | G | Member of RPA complex. | **+** |  |
| RPA-bound telomeres | S | A non-sequence specific ssDNA binding complex of RPA1, RPA2 and RPA3. Replaces POT1 and recruits ATR to ssDNA at telomeres. | **+** |  |
| HNRNPA1 | G | Displaces RPA from ssDNA, promoting the RPA-POT1 switch during the cell cycle. | **-** |  |
| ATR | G | Ser/Thr kinase, DNA damage sensing and checkpoint protein. Phosphorylates CHEK1. Activates HR at telomeres upon recruitment by RPA (Flynn et al., 2012). Inhibition reduces ALT (Flynn et al., 2015). Inhibition does not universally kill ALT cells (Deeg et al., 2016). Disruption of ATR and CHEK1 inhibits recruitment of HOP2 to telomeres (Dilley et al., 2016). | **+** | (Flynn et al., 2012), (Flynn et al., 2015), (Deeg et al., 2016), (Dilley et al., 2016) |
| CHEK1 | G | Disruption of ATR and CHEK1 inhibits recruitment of HOP2 to telomeres (Dilley et al., 2016). | **+** | (Dilley et al., 2016) |
| RAD51 | G | Is recruited to RPA-bound telomeres. Promotes homology search and strand-exchange (Cho et al., 2014). May supppress c-circle formation (Jia-Min Zhang et al., 2019). | **+** | (Cho et al., 2014),  (Jia-Min Zhang et al., 2019) |
| PSMC3IP (HOP2) | G | In complex with MND1 and together with RAD51 facilitates long-range DNA movement, homology search and strand exchange. | **+** | (Cho et al., 2014) |
| MND1 | G | In complex with HOP2 and together with RAD51 facilitates long-range DNA movement, homology search and strand exchange. | **+** |  |
| HOP2-MND1 | C | Together with RAD51 facilitates long-range DNA movement, homology search and strand exchange. | **+** |  |
| RAD52 | G | Required for ALT in some cases, but not for C-circle formation. Promotes strand invasion and telomeric D-loop formation even in the presence of RPA (Jia-Min Zhang et al., 2019). May promote annealing of resected single-stranded telomeres to potential templates (Min et al., 2019). | **+** | (Jia-Min Zhang et al., 2019), (Min et al., 2019) |
|  |  |  |  |  |
| Template directed synthesis | | | | |
| *Polymerase directed synthesis of the telomeric G- and C- strands* | | | | |
| POLD3 | G | Subunit of DNA polymerase delta. Required for synthesis of G- and C- strands. | **+** | (Dilley et al., 2016) |
| PCNA | G | Cofactor of DNA polymerase delta. Part of DNA-clamp complex. Interacts with POLD3 to recruit polymerase delta to ALT telomeres. | **+** |  |
| RFC1 | G | DNA-dependent ATPase. Part of RFC clamp loading complex, binds to 3' primers and facilitates DNA synthesis at both strands. Senses damaged telomeres and facilitates PCNA-POLD3 loading. | **+** |  |
| clamp loading complex | C | Рequired for PCNA–POLD3 telomere localization | **+** |  |
| PNCA clamp | C | Processive synthesis and strand displacement | **+** |  |
| Holiday Junction (HJ) processing | | | | |
| *HJ dissolution with telomere synthesis* | | | | |
| MRN | C | Acts at first stages of DSBR: detects and tethers DSBs, activates damage response. May inhibit NHEJ by promoting generation of 3' overhang after leading strand synthesis (Dimitrova and de Lange, 2009). Depletion or sequestration of MRN complex supresses the ALT pathway (Clynes et al., 2015). | **+** | (Dimitrova and de Lange, 2009), (Clynes et al., 2015) |
|  |  |  |  |  |
| MRE11 | G | Member of MRN complex. An endonuclease. | **+** | (Lafrance-Vanasse et al., 2015) |
| RAD50 | G | Member of MRN complex. An ATPase. | **+** |  |
| NBN (NBS1) | G | Member of MRN complex. An adapter protein. | **+** |  |
| SP100 | G | Sequesters MRN away from ABPs. Suppresses ALT. | **-** | (Jiang et al., 2005) |
| BTR | C | Promotes long-tract telomere extension followed by 5'-3' end resection and telomeric dissolution. | **+** | (Sobinoff et al., 2017), (Min et al., 2019) |
| BLM | G | Member of BTR complex. RecQ DNA helicase. | **+** | (Sobinoff et al., 2017) |
| TOP3A | G | Member of BTR complex. DNA topoisomerase. | **+** |  |
| RMI1 | G | Member of BTR complex. Accessory protein. | **+** |  |
| RMI2 | G | Member of BTR complex. Accessory protein. | **+** |  |
| *HJ resolution preventing telomere synthesis* | | | | |
| ERRC1/4 | C | Promotes telomeric resolution, represses association of POLD3 with telomeres and prevents telomere synthesis by resolving recombination intermediates. | **-** | (Zhu et al., 2003) |
| SLX1 | G | Holiday Junction resolvase. | **-** | (Sobinoff et al., 2017) |
| SLX4 | G | Scaffold for endonucleases' assembly. Recruits endonucleases. | **-** |  |
| SLX1A | G | Catalytic subunit of SLX4-SLX1 | **-** |  |
| SLX1B | G | Catalytic subunit of SLX4-SLX1 | **-** |  |
| ERCC1 | G | Forms a heterodimer with ERCC4 and supresses recombination with interstitial telomeric sequences. | **-** | (Zhu et al., 2003) |
| ERCC4 | G | Forms a heterodimer with ERCC1 and supresses recombination with interstitial telomeric sequences. | **-** |  |

^a^ Node types: G – gene, C – complex, I – isoform.

^b^ Effect types: ‘-’ - inhibitory effect, ‘+’ – activating effect

## Table S2. Functional role of the TEL pathway nodes.

| **Name (alias)** | **Type** | **Description** | **Effect** | **Reference** |
| --- | --- | --- | --- | --- |
| Nuclear TERT (TERT activation and recruitment) | | | | |
| *TERT expression, post-transcriptional modifications and factors involved in recruitment of the active hTERT protein to the nucleus* | | | | |
| TERT | G | The catalytic subunit of telomerase | **+** | (Cohen et al., 2007) |
| KPNA1 | G | Importin alpha 5. Interacts with nuclear localization signal of hTERT and promotes its nuclear import. | **+** | (Jeong et al., 2015) |
| RAN | G | GTPase that affects disassembly of importin-cargo complexes and is important for importin alpha 5 mediated nuclear import of hTERT. | **+** |  |
| IPO7 | G | Importin alpha 7. Interacts with the C-terminal nuclear localization signal of hTERT and promotes its alternative nuclear import. | **+** | (Frohnert et al., 2014) |
| RANBP2 | G | A nuclear pore complex member. Plays an important role in importin alpha 7 mediated nuclear import of hTERT. | **+** |  |
| HSP90 | I | A chaperone that assures proper hTERT conformation to promote its nuclear import. | **+** | (Jeong et al., 2015) |
| HSP90AA1 | G | HSP90 isoform | **+** |  |
| HSP90AA2 | G | HSP90 isoform | **+** |  |
| HSP90AB1 | G | HSP90 isoform | **+** |  |
| PTGES3 | G | p23, a co-chaperone that binds HSP90 dimer and stabilizes the HSP90-hTERT complex. | **+** |  |
| STUB1 | G | CHIP, a ubiquitine ligase that marks cytoplasmic hTERT for degradation (Lee et al., 2010). | **-** | (Lee et al., 2010) |
| hTR maturation | | | | |
| TERC expression, and opposing processes of degradation of the transcripts or stabilitzation and maturation into functional hTR template | | | | |
| TERC | G | The RNA template (hTR) of telomerase | **+** | (Cohen et al., 2007), (Cayuela et al., 2005) |
| PARN | G | Ribonuclease that removes oligo(A) tails from nascent hTR, adds poly(A) tails and promotes hTR maturation. | **+** | (Moon et al., 2015), (Boyraz et al., 2016) |
| TRAMP | C | A polyadenylation complex consisting of PAPD5 (TENT4B), MTR4 (MTREX) and AIR2 (ZCCHC7). Adds oligo(A) tails to nascent hTRs and marks them for degradation by the exosomes. | **-** | (Boyraz et al., 2016) |
| TENT4B (PAPD5) | G | Catalytic subunit of the TRAMP complex. | **-** | (Tseng et al., 2015), (Moon et al., 2015) |
| MTREX (MTR4) | G | A helicase, part of the TRAMP complex. | **-** | (Tseng et al., 2015) |
| ZCCHC7 (AIR2) | G | RNA-binding factor, member of the TRAMP complex. | **-** |  |
| DKC1-NOP10-NHP2-NAF1 | C | This complex binds hTR contranslationally, protects it from degradation and leads to maturation in Cajal bodies. | **+** | (Schmidt and Cech, 2015) |
| DKC1 | G | Key component of telomerase; part of hTR maturation complex DKC1-NOP10-NHP2-NAF1 | **+** |  |
| NOP10 | G | Small nucleolar ribonucleoprotein. Member of DKC1-NOP10-NHP2-NAF1 complex. | **+** |  |
| NHP2 | G | Small nucleolar ribonucleoprotein. Member of DKC1-NOP10-NHP2-NAF1 complex. | **+** |  |
| NAF1 | G | Ribonucleoprotein assembly factor. Member of DKC1-NOP10-NHP2-NAF1 complex. | **+** |  |
| GAR1 | G | Ribonucleoprotein assembly factor: replaces NAF1 during ribonucleoprotein complex maturation. Member of DKC1-NOP10-NHP2-GAR1 complex. | **+** |  |
| WRAP53 | G | RNA chaperone that promotes proper localization of hTR to Cajal bodies. Also may assist in hTR folding after telomerase assembly. | **+** | (Chen et al., 2018) |
| Telomerase assembly | | | | |
| *Main components of the telomerase complex and additional factors promoting its assembly* | | | | |
| TERT | G | The catalytic subunit of telomerase | **+** | (Cohen et al., 2007) |
| TERC | G | The RNA template (hTR) of telomerase | **+** | (Cohen et al., 2007), (Cayuela et al., 2005) |
| DKC1 | G | Core component of telomerase: stabilizes the complex by binding to hTR. | **+** | (Schmidt and Cech, 2015) |
| Pontin/Reptin | C | ATPases that are shown to act together and ensure proper telomerase assembly. | **+** | (Venteicher et al., 2008) |
| RUVBL1 | G | Pontin: part of pontin/reptin complex. Interacts directly with hTERT. | **+** |  |
| RUVBL2 | G | Reptin: part of pontin/reptin complex. | **+** |  |
| Recruitment to telomeres and synthesis | | | | |
| *Factors recruiting the telomerase assembly to the telomeres and promoting telomere synthesis* | | | | |
| CST | C | Has dual role in the pathway. Recruits telomerase to telomeres, but limits its presence to one extension cycle only, competing with POT1-TPP1 for telomere binding. Recruits polymerase alpha to complete telomere synthesis. | **+** | (Chen et al., 2012) |
| CTC1 | G | Member of CST complex. | **+** |  |
| STN1 | G | Member of CST complex. Stimulates the activity of DNA polymerase alpha. | **+** |  |
| TEN1 | G | Member of CST complex. | **+** |  |
| Telomerase | C | Enzyme that counteracts telomeric DNA shortening | **+** |  |

^a^ Node types: G – gene, C – complex, I – isoform.

^b^ Effect types: ‘-’ - inhibitory effect, ‘+’ – activating effect

# Pathway Signal Flow algorithm

For pathway activity estimation from gene expression data we have used the Pathway Signal Flow (PSF) algorithm, implemented in the *PSFC* app for Cytoscape, v1.1.8 (Nersisyan et al., 2015b). In this particular study, we have made use of a higher level Cytoscape app, *TMM* v0.8 that uses PSFC as a dependency. It compares TMM pathway activation patterns with experimental annotations, uses PSFC for pathway activity computation and it also produces reports for TMM phenotype comparison across samples.The app (optionally) computes the fold change (FC) of each gene by taking the ratio of its expression to the average expression across the samples of the respective data set.

The PSF algorithm (Arakelyan et al., 2013; Nersisyan et al., 2015b, 2016) computes the strength of the signal propagated from the pathway inputs to the outputs through pairwise interactions between nodes, based on their fold change (FC) expression values. In this study, missing values were assigned an FC value of 1. For each source-target interaction, the FC values were multiplied for edges of type *activation* (FC_source_ * FC_target_) and inversely multiplied for edges of type *inhibition* (1/FC_source_ * FC_target_). The signal propagation starts from input nodes, spreads through the intermediate nodes and arrives at the sink nodes (labeled “ALT” and “TEL”, respectively). The PSF scores at the sink nodes reflect the overall activity of the pathways (Figure S1).

The PSF threshold for considering the pathway activity as high is defined based on the gene expression data applied. This approach is dictated by the diversity of technologies used for gene expression measurements as well as variety of tools used for its preprocessing. However, in well balanced datasets, with relatively equal counts of ALT and TEL samples the positive log PSF values would indicate high activity of the corresponding pathway.

The product of multiple signals from many sources was assigned as the signal at the target node (the “multiplication” option in PSFC). PSFC also allows for assigning explicit functions to deal with multiple incoming edges onto specific nodes. We have assigned the function “min” to nodes combining complex subunits (as the gene with minimum expression defines the activity of the complex), and the function “max” to homologous genes, where it is unknown which transcript plays the described role (and assumed that the most expressed one should), and finally we assigned the function “sum” to the linker between two hTERT nuclear import pathways, as each of them independently contribute to the protein’s entry to the nucleus.


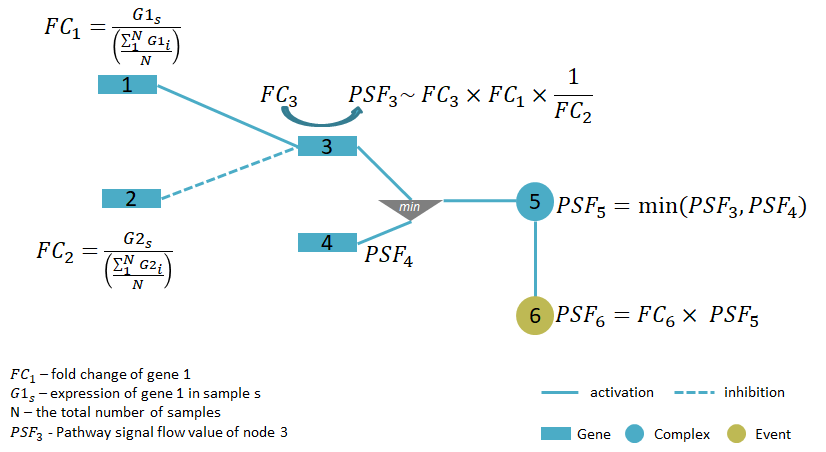


Figure S1. Signal propagation with the pathway signal flow algorithm.

The fold change (FC) values for each gene are computed as its expression in the given sample relative to its mean expression across the samples. After initial assignment of FC values, the pathway signal flow (PSF) values are computed by multiplying the source and target values (PSF if already computed or FC if the node doesn’t have upstream interactors) on the edges of type activation, or performing inverse multiplication on the edges of type inhibition. The operator nodes (min) modulate the PSF values by setting a certain rule on multiple input signals (in this case: the minimum value of the two upstream PSF values on the node of type Complex).


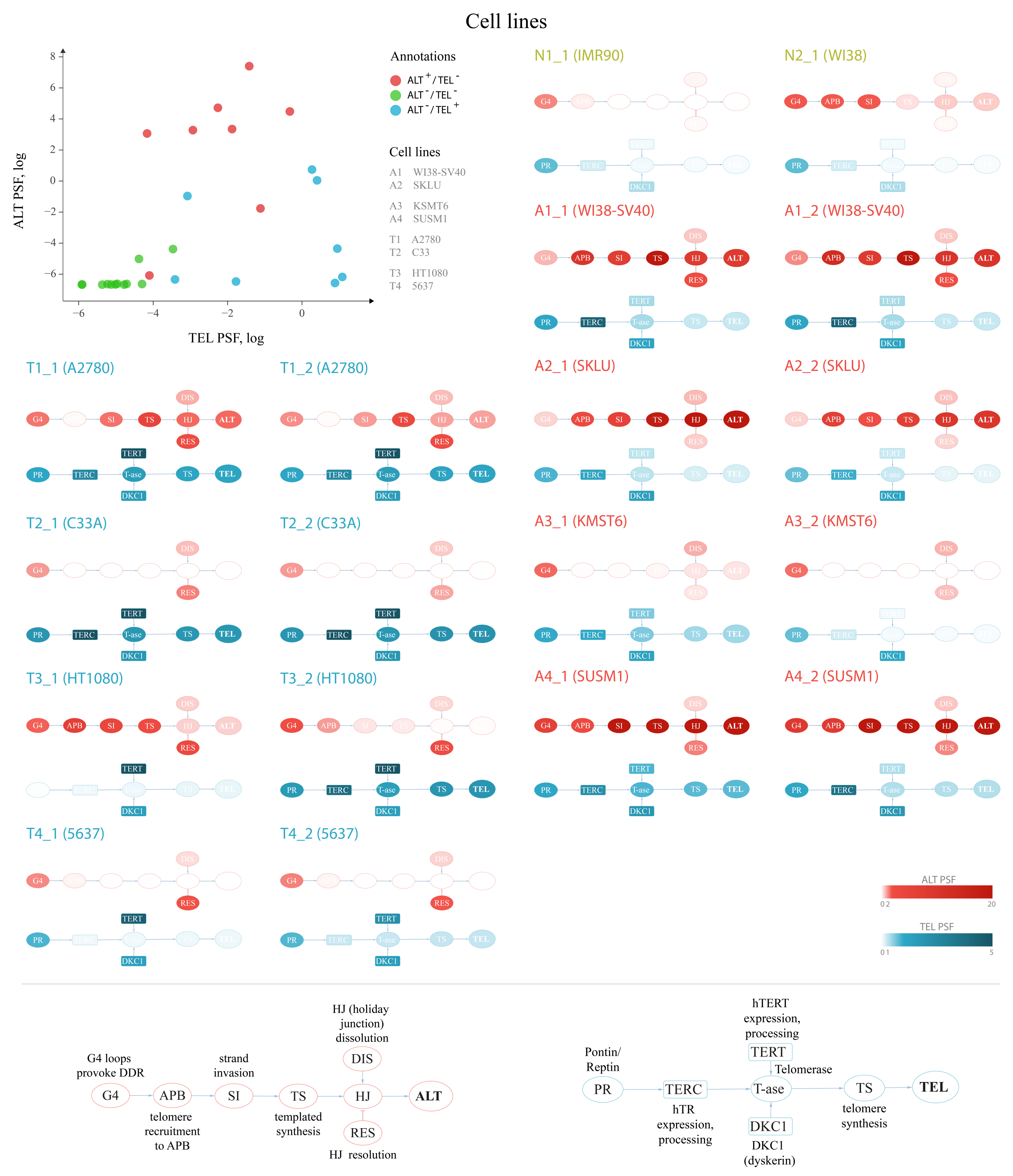


Figure S2. Cell line specific pathway activation patterns at the level of individual pathway branches.

Top left: 2D plots with samples placed according to the TEL (x axis) and the ALT (y axis) pathway activity PSF values. The samples are colored according to the experimental TMM annotations. Technical replicates are distinguished with _1 and _2 suffices. Horizontal and vertical dash lines separate ALT^+^ from ALT^‑^ and TEL^+^ from TEL^-^ experimentally annotated samples based on support vector machine classification on the ALT and the TEL PSF values. Other panels: Pathway activation patterns at the level of branches in each sample. Bottom: node abbreviations in the pathways.


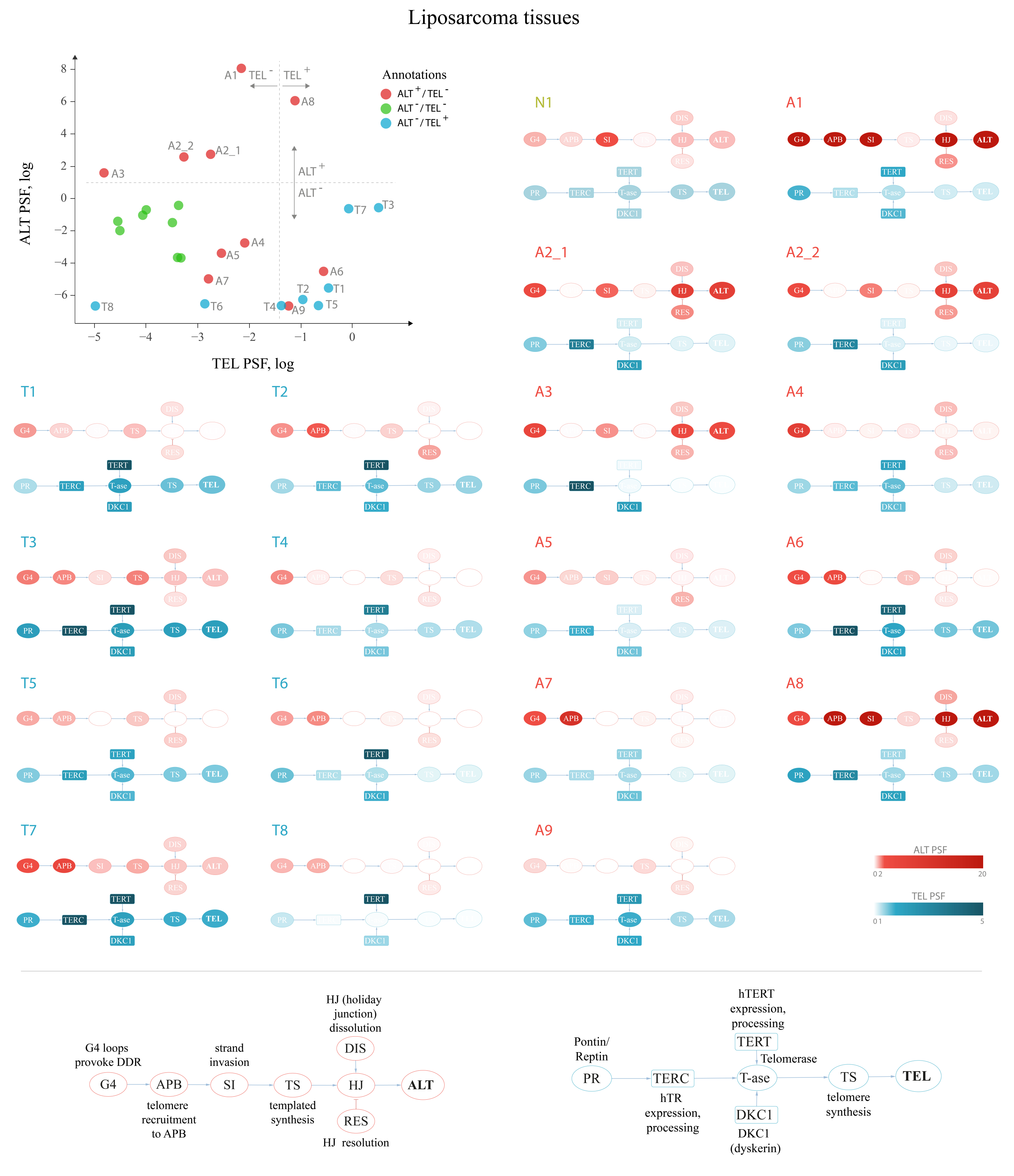


Figure S3. Tissue specific pathway activation patterns at the level of individual pathway branches.

Top left: 2D plots with samples placed according to the TEL (x axis) and the ALT (y axis) pathway activity PSF values. The samples are colored according to the experimental TMM annotations. Technical replicates are distinguished with _1 and _2 suffices. Horizontal and vertical dash lines separate ALT^+^ from ALT^‑^ and TEL^+^ from TEL^-^ experimentally annotated samples based on support vector machine classification on the ALT and the TEL PSF values. Other panels: Pathway activation patterns at the level of branches in each sample. Bottom: node abbreviations in the pathways.


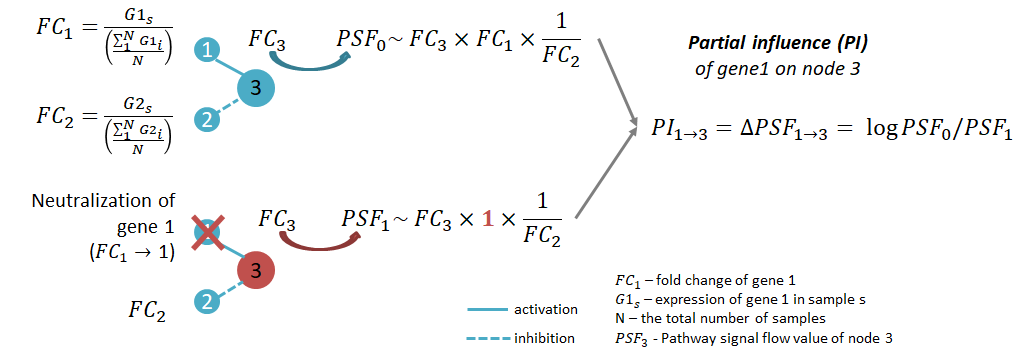


Figure S4. A toy example of computation of the partial influence (PI) of a given source node on the PSF activity of the target node.

Top: computation of the PSF activity of the node 3 (PSF_0_), given the fold change (FC) values of the node 1 and 2. Bottom: PSF activity of the node 3 (PSF_1_) after setting the fold change of the node 1 to FC = 1, thus neutralizing its influence. The difference between the PSF activity of the node 3 before and after neutralizing the influence of the node 1 is the partial influence (PI) of the node 1 on node 3 activity.


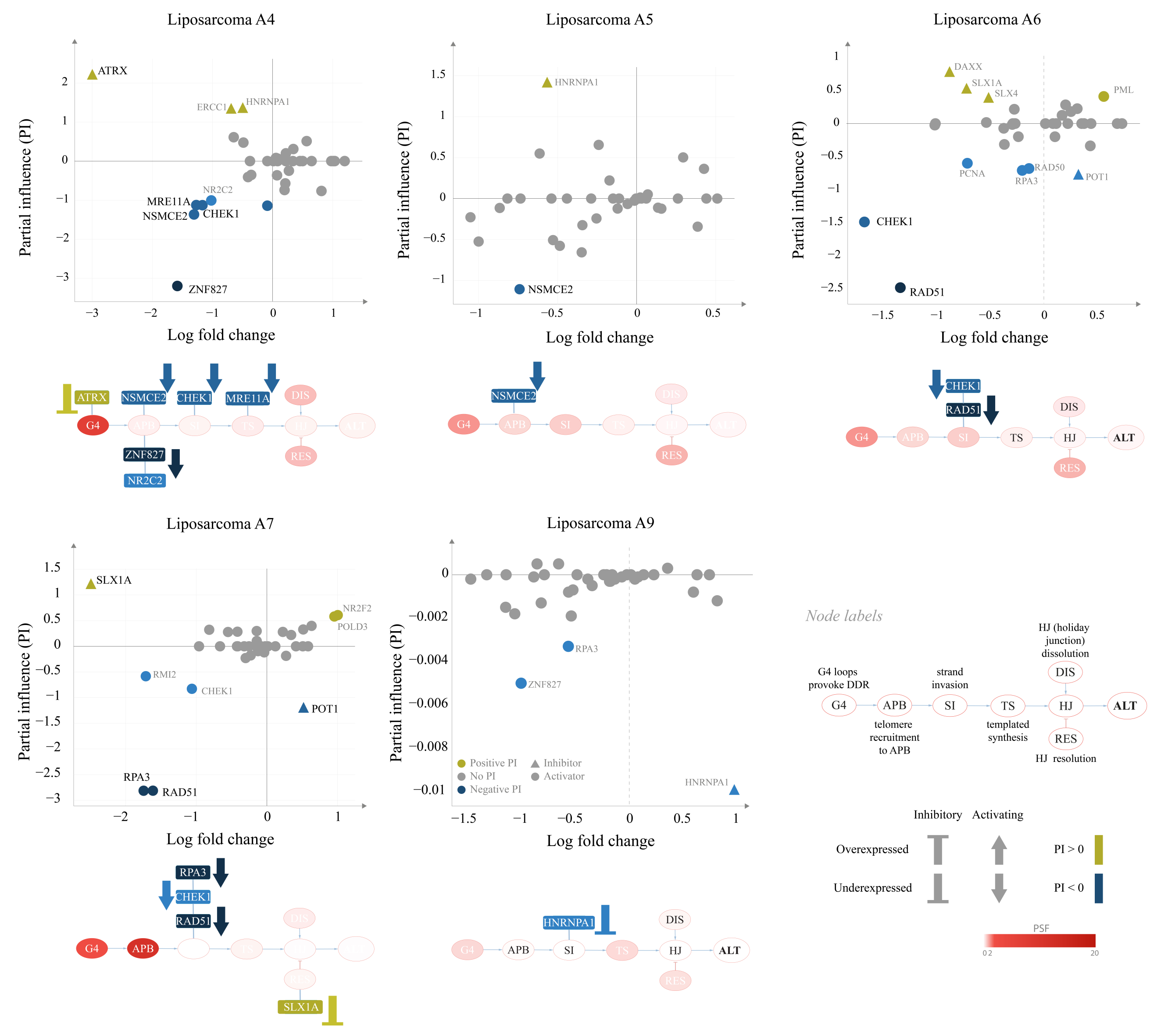


Figure S5. Partial influence of nodes on the ALT pathway activity of the A5 and A9 liposarcoma tissues.

Partial influence (PI) of each node is computed as the difference of the TEL node PSF value when the node is set to a fold change (FC) value of 1. The most influential nodes are shown in the context of the pathway branches.


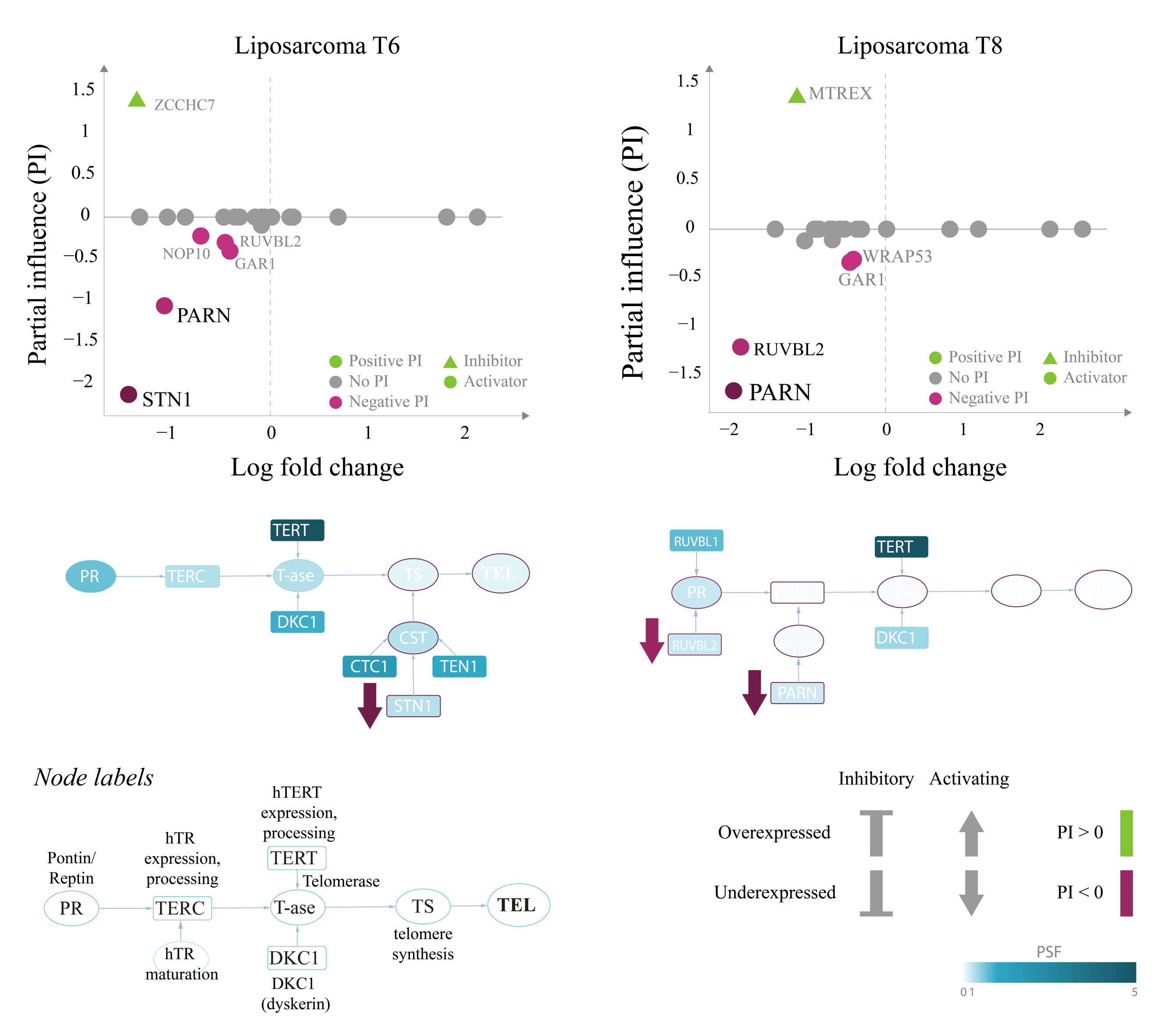
**Figure S6. Partial influence of nodes on the** **TEL** **pathway activity of the T6 and T8 liposarcoma tissues.**

Partial influence (PI) of each node is computed as the difference of the TEL node PSF value when the node is set to a fold change (FC) value of 1. The most influential nodes are shown in the context of the pathway branches.

Table S3. Genes from the TelNet database that are marked as either having a role in ALT or Telomerase-mediated telomere maintenance pathways.

| **Entrez** | **Symbol** | **TMM** |  | **Entrez** | **Symbol** | **TMM** |
| --- | --- | --- | --- | --- | --- | --- |
| 52 | ACP1 | ALT |  | 546 | ATRX | Telomerase-mediated |
| 367 | AR | ALT |  | 553 | AVPR1B | Telomerase-mediated |
| 641 | BLM | ALT |  | 624 | BDKRB2 | Telomerase-mediated |
| 655 | BMP7 | ALT |  | 815 | CAMK2A | Telomerase-mediated |
| 675 | BRCA2 | ALT |  | 908 | CCT6A | Telomerase-mediated |
| 818 | CAMK2G | ALT |  | 924 | CD7 | Telomerase-mediated |
| 860 | RUNX2 | ALT |  | 1030 | CDKN2B | Telomerase-mediated |
| 1026 | CDKN1A | ALT |  | 1111 | CHEK1 | Telomerase-mediated |
| 1050 | CEBPA | ALT |  | 1198 | CLK3 | Telomerase-mediated |
| 1051 | CEBPB | ALT |  | 1432 | MAPK14 | Telomerase-mediated |
| 1763 | DNA2 | ALT |  | 1457 | CSNK2A1 | Telomerase-mediated |
| 1786 | DNMT1 | ALT |  | 1499 | CTNNB1 | Telomerase-mediated |
| 1869 | E2F1 | ALT |  | 1616 | DAXX | Telomerase-mediated |
| 2072 | ERCC4 | ALT |  | 1633 | DCK | Telomerase-mediated |
| 2073 | ERCC5 | ALT |  | 1736 | DKC1 | Telomerase-mediated |
| 2175 | FANCA | ALT |  | 1740 | DLG2 | Telomerase-mediated |
| 2237 | FEN1 | ALT |  | 1742 | DLG4 | Telomerase-mediated |
| 2547 | XRCC6 | ALT |  | 1760 | DMPK | Telomerase-mediated |
| 3020 | H3F3A | ALT |  | 1841 | DTYMK | Telomerase-mediated |
| 3104 | ZBTB48 | ALT |  | 1870 | E2F2 | Telomerase-mediated |
| 3178 | HNRNPA1 | ALT |  | 1871 | E2F3 | Telomerase-mediated |
| 3183 | HNRNPC | ALT |  | 1874 | E2F4 | Telomerase-mediated |
| 3190 | HNRNPK | ALT |  | 1875 | E2F5 | Telomerase-mediated |
| 3192 | HNRNPU | ALT |  | 1876 | E2F6 | Telomerase-mediated |
| 3659 | IRF1 | ALT |  | 1907 | EDN2 | Telomerase-mediated |
| 3725 | JUN | ALT |  | 2043 | EPHA4 | Telomerase-mediated |
| 3978 | LIG1 | ALT |  | 2099 | ESR1 | Telomerase-mediated |
| 4084 | MXD1 | ALT |  | 2100 | ESR2 | Telomerase-mediated |
| 4088 | SMAD3 | ALT |  | 2113 | ETS1 | Telomerase-mediated |
| 4150 | MAZ | ALT |  | 2114 | ETS2 | Telomerase-mediated |
| 4217 | MAP3K5 | ALT |  | 2115 | ETV1 | Telomerase-mediated |
| 4221 | MEN1 | ALT |  | 2118 | ETV4 | Telomerase-mediated |
| 4361 | MRE11 | ALT |  | 2130 | EWSR1 | Telomerase-mediated |
| 4683 | NBN | ALT |  | 2242 | FES | Telomerase-mediated |
| 5105 | PCK1 | ALT |  | 2261 | FGFR3 | Telomerase-mediated |
| 5156 | PDGFRA | ALT |  | 2264 | FGFR4 | Telomerase-mediated |
| 5307 | PITX1 | ALT |  | 2313 | FLI1 | Telomerase-mediated |
| 5371 | PML | ALT |  | 2596 | GAP43 | Telomerase-mediated |
| 5422 | POLA1 | ALT |  | 2735 | GLI1 | Telomerase-mediated |
| 5424 | POLD1 | ALT |  | 2736 | GLI2 | Telomerase-mediated |
| 5425 | POLD2 | ALT |  | 3091 | HIF1A | Telomerase-mediated |
| 5426 | POLE | ALT |  | 3181 | HNRNPA2B1 | Telomerase-mediated |
| 5427 | POLE2 | ALT |  | 3184 | HNRNPD | Telomerase-mediated |
| 5429 | POLH | ALT |  | 3204 | HOXA7 | Telomerase-mediated |
| 5468 | PPARG | ALT |  | 3320 | HSP90AA1 | Telomerase-mediated |
| 5557 | PRIM1 | ALT |  | 3326 | HSP90AB1 | Telomerase-mediated |
| 5558 | PRIM2 | ALT |  | 3417 | IDH1 | Telomerase-mediated |
| 5599 | MAPK8 | ALT |  | 3654 | IRAK1 | Telomerase-mediated |
| 5888 | RAD51 | ALT |  | 3717 | JAK2 | Telomerase-mediated |
| 5889 | RAD51C | ALT |  | 3727 | JUND | Telomerase-mediated |
| 5906 | RAP1A | ALT |  | 3937 | LCP2 | Telomerase-mediated |
| 5925 | RB1 | ALT |  | 4149 | MAX | Telomerase-mediated |
| 5981 | RFC1 | ALT |  | 4214 | MAP3K1 | Telomerase-mediated |
| 5982 | RFC2 | ALT |  | 4215 | MAP3K3 | Telomerase-mediated |
| 5983 | RFC3 | ALT |  | 4216 | MAP3K4 | Telomerase-mediated |
| 5984 | RFC4 | ALT |  | 4233 | MET | Telomerase-mediated |
| 5985 | RFC5 | ALT |  | 4286 | MITF | Telomerase-mediated |
| 6093 | ROCK1 | ALT |  | 4436 | MSH2 | Telomerase-mediated |
| 6119 | RPA3 | ALT |  | 4598 | MVK | Telomerase-mediated |
| 6199 | RPS6KB2 | ALT |  | 4602 | MYB | Telomerase-mediated |
| 6259 | RYK | ALT |  | 4609 | MYC | Telomerase-mediated |
| 6612 | SUMO3 | ALT |  | 4613 | MYCN | Telomerase-mediated |
| 6613 | SUMO2 | ALT |  | 4751 | NEK2 | Telomerase-mediated |
| 6615 | SNAI1 | ALT |  | 4773 | NFATC2 | Telomerase-mediated |
| 6670 | SP3 | ALT |  | 4790 | NFKB1 | Telomerase-mediated |
| 6672 | SP100 | ALT |  | 4846 | NOS3 | Telomerase-mediated |
| 6714 | SRC | ALT |  | 4914 | NTRK1 | Telomerase-mediated |
| 6886 | TAL1 | ALT |  | 4931 | NVL | Telomerase-mediated |
| 6932 | TCF7 | ALT |  | 5079 | PAX5 | Telomerase-mediated |
| 7011 | TEP1 | ALT |  | 5287 | PIK3C2B | Telomerase-mediated |
| 7025 | NR2F1 | ALT |  | 5395 | PMS2 | Telomerase-mediated |
| 7026 | NR2F2 | ALT |  | 5570 | PKIB | Telomerase-mediated |
| 7153 | TOP2A | ALT |  | 5576 | PRKAR2A | Telomerase-mediated |
| 7156 | TOP3A | ALT |  | 5577 | PRKAR2B | Telomerase-mediated |
| 7157 | TP53 | ALT |  | 5588 | PRKCQ | Telomerase-mediated |
| 7158 | TP53BP1 | ALT |  | 5589 | PRKCSH | Telomerase-mediated |
| 7161 | TP73 | ALT |  | 5594 | MAPK1 | Telomerase-mediated |
| 7181 | NR2C1 | ALT |  | 5595 | MAPK3 | Telomerase-mediated |
| 7182 | NR2C2 | ALT |  | 5609 | MAP2K7 | Telomerase-mediated |
| 7323 | UBE2D3 | ALT |  | 5611 | DNAJC3 | Telomerase-mediated |
| 7329 | UBE2I | ALT |  | 5793 | PTPRG | Telomerase-mediated |
| 7341 | SUMO1 | ALT |  | 5879 | RAC1 | Telomerase-mediated |
| 7345 | UCHL1 | ALT |  | 5970 | RELA | Telomerase-mediated |
| 7421 | VDR | ALT |  | 6023 | RMRP | Telomerase-mediated |
| 7486 | WRN | ALT |  | 6117 | RPA1 | Telomerase-mediated |
| 7490 | WT1 | ALT |  | 6635 | SNRPE | Telomerase-mediated |
| 7517 | XRCC3 | ALT |  | 6774 | STAT3 | Telomerase-mediated |
| 7520 | XRCC5 | ALT |  | 6776 | STAT5A | Telomerase-mediated |
| 7593 | MZF1 | ALT |  | 6787 | NEK4 | Telomerase-mediated |
| 7913 | DEK | ALT |  | 6790 | AURKA | Telomerase-mediated |
| 8289 | ARID1A | ALT |  | 6829 | SUPT5H | Telomerase-mediated |
| 8379 | MAD1L1 | ALT |  | 6934 | TCF7L2 | Telomerase-mediated |
| 8491 | MAP4K3 | ALT |  | 7012 | TERC | Telomerase-mediated |
| 8553 | BHLHE40 | ALT |  | 7015 | TERT | Telomerase-mediated |
| 8932 | MBD2 | ALT |  | 7020 | TFAP2A | Telomerase-mediated |
| 9839 | ZEB2 | ALT |  | 7021 | TFAP2B | Telomerase-mediated |
| 10038 | PARP2 | ALT |  | 7023 | TFAP4 | Telomerase-mediated |
| 10111 | RAD50 | ALT |  | 7203 | CCT3 | Telomerase-mediated |
| 10320 | IKZF1 | ALT |  | 7360 | UGP2 | Telomerase-mediated |
| 10365 | KLF2 | ALT |  | 7515 | XRCC1 | Telomerase-mediated |
| 10411 | RAPGEF3 | ALT |  | 7525 | YES1 | Telomerase-mediated |
| 10445 | MCRS1 | ALT |  | 7707 | ZNF148 | Telomerase-mediated |
| 10574 | CCT7 | ALT |  | 7849 | PAX8 | Telomerase-mediated |
| 10575 | CCT4 | ALT |  | 8091 | HMGA2 | Telomerase-mediated |
| 10576 | CCT2 | ALT |  | 8518 | IKBKAP | Telomerase-mediated |
| 10635 | RAD51AP1 | ALT |  | 8550 | MAPKAPK5 | Telomerase-mediated |
| 10664 | CTCF | ALT |  | 8573 | CASK | Telomerase-mediated |
| 10714 | POLD3 | ALT |  | 8576 | STK16 | Telomerase-mediated |
| 10715 | CERS1 | ALT |  | 8607 | RUVBL1 | Telomerase-mediated |
| 11176 | BAZ2A | ALT |  | 8651 | SOCS1 | Telomerase-mediated |
| 11335 | CBX3 | ALT |  | 8658 | TNKS | Telomerase-mediated |
| 22928 | SEPHS2 | ALT |  | 8859 | STK19 | Telomerase-mediated |
| 22948 | CCT5 | ALT |  | 8986 | RPS6KA4 | Telomerase-mediated |
| 23028 | KDM1A | ALT |  | 8999 | CDKL2 | Telomerase-mediated |
| 23137 | SMC5 | ALT |  | 9019 | MPZL1 | Telomerase-mediated |
| 23293 | SMG6 | ALT |  | 9024 | BRSK2 | Telomerase-mediated |
| 23515 | MORC3 | ALT |  | 9212 | AURKB | Telomerase-mediated |
| 23649 | POLA2 | ALT |  | 9252 | RPS6KA5 | Telomerase-mediated |
| 51564 | HDAC7 | ALT |  | 9314 | KLF4 | Telomerase-mediated |
| 51750 | RTEL1 | ALT |  | 9475 | ROCK2 | Telomerase-mediated |
| 54107 | POLE3 | ALT |  | 9878 | TOX4 | Telomerase-mediated |
| 54984 | PINX1 | ALT |  | 10020 | GNE | Telomerase-mediated |
| 55183 | RIF1 | ALT |  | 10270 | AKAP8 | Telomerase-mediated |
| 55226 | NAT10 | ALT |  | 10694 | CCT8 | Telomerase-mediated |
| 55300 | PI4K2B | ALT |  | 10725 | NFAT5 | Telomerase-mediated |
| 56655 | POLE4 | ALT |  | 10728 | PTGES3 | Telomerase-mediated |
| 56848 | SPHK2 | ALT |  | 10738 | RFPL3 | Telomerase-mediated |
| 56849 | TCEAL7 | ALT |  | 10746 | MAP3K2 | Telomerase-mediated |
| 57804 | POLD4 | ALT |  | 10783 | NEK6 | Telomerase-mediated |
| 60496 | AASDHPPT | ALT |  | 10842 | PPP1R17 | Telomerase-mediated |
| 60561 | RINT1 | ALT |  | 10856 | RUVBL2 | Telomerase-mediated |
| 64781 | CERK | ALT |  | 10892 | MALT1 | Telomerase-mediated |
| 65220 | NADK | ALT |  | 10898 | CPSF4 | Telomerase-mediated |
| 79648 | MCPH1 | ALT |  | 10971 | YWHAQ | Telomerase-mediated |
| 79677 | SMC6 | ALT |  | 11200 | CHEK2 | Telomerase-mediated |
| 80119 | PIF1 | ALT |  | 11221 | DUSP10 | Telomerase-mediated |
| 80169 | CTC1 | ALT |  | 11284 | PNKP | Telomerase-mediated |
| 80198 | MUS81 | ALT |  | 22983 | MAST1 | Telomerase-mediated |
| 80724 | ACAD10 | ALT |  | 23043 | TNIK | Telomerase-mediated |
| 84126 | ATRIP | ALT |  | 23326 | USP22 | Telomerase-mediated |
| 84464 | SLX4 | ALT |  | 23552 | CDK20 | Telomerase-mediated |
| 84787 | KMT5C | ALT |  | 25842 | ASF1A | Telomerase-mediated |
| 152485 | ZNF827 | ALT |  | 25849 | PARM1 | Telomerase-mediated |
| 201516 | ZSCAN4 | ALT |  | 25865 | PRKD2 | Telomerase-mediated |
| 286053 | NSMCE2 | ALT |  | 25913 | POT1 | Telomerase-mediated |
| 348825 | TPRXL | ALT |  | 26054 | SENP6 | Telomerase-mediated |
| 493861 | EID3 | ALT |  | 26060 | APPL1 | Telomerase-mediated |
| 548593 | SLX1A | ALT |  | 26585 | GREM1 | Telomerase-mediated |
| 100134934 | TEN1 | ALT |  | 29094 | LGALSL | Telomerase-mediated |
| 100421108 | LOC100421108 | ALT |  | 50485 | SMARCAL1 | Telomerase-mediated |
| 100507436 | MICA | ALT |  | 51378 | ANGPT4 | Telomerase-mediated |
|  |  |  |  | 51765 | STK26 | Telomerase-mediated |
|  |  |  |  | 53340 | SPA17 | Telomerase-mediated |
|  |  |  |  | 54433 | GAR1 | Telomerase-mediated |
|  |  |  |  | 55120 | FANCL | Telomerase-mediated |
|  |  |  |  | 55135 | WRAP53 | Telomerase-mediated |
|  |  |  |  | 55164 | SHQ1 | Telomerase-mediated |
|  |  |  |  | 55197 | RPRD1A | Telomerase-mediated |
|  |  |  |  | 55505 | NOP10 | Telomerase-mediated |
|  |  |  |  | 55589 | BMP2K | Telomerase-mediated |
|  |  |  |  | 55651 | NHP2 | Telomerase-mediated |
|  |  |  |  | 55723 | ASF1B | Telomerase-mediated |
|  |  |  |  | 55869 | HDAC8 | Telomerase-mediated |
|  |  |  |  | 57144 | PAK5 | Telomerase-mediated |
|  |  |  |  | 57410 | SCYL1 | Telomerase-mediated |
|  |  |  |  | 64799 | IQCH | Telomerase-mediated |
|  |  |  |  | 79366 | HMGN5 | Telomerase-mediated |
|  |  |  |  | 79934 | COQ8B | Telomerase-mediated |
|  |  |  |  | 79977 | GRHL2 | Telomerase-mediated |
|  |  |  |  | 80351 | TNKS2 | Telomerase-mediated |
|  |  |  |  | 84152 | PPP1R1B | Telomerase-mediated |
|  |  |  |  | 92335 | STRADA | Telomerase-mediated |
|  |  |  |  | 92345 | NAF1 | Telomerase-mediated |
|  |  |  |  | 117283 | IP6K3 | Telomerase-mediated |
|  |  |  |  | 140609 | NEK7 | Telomerase-mediated |
|  |  |  |  | 140690 | CTCFL | Telomerase-mediated |
|  |  |  |  | 170506 | DHX36 | Telomerase-mediated |
|  |  |  |  | 225689 | MAPK15 | Telomerase-mediated |
|  |  |  |  | 256281 | NUDT14 | Telomerase-mediated |
|  |  |  |  | 283455 | KSR2 | Telomerase-mediated |
|  |  |  |  | 284086 | NEK8 | Telomerase-mediated |
|  |  |  |  | 285220 | EPHA6 | Telomerase-mediated |
|  |  |  |  | 340061 | TMEM173 | Telomerase-mediated |
|  |  |  |  | 440275 | EIF2AK4 | Telomerase-mediated |


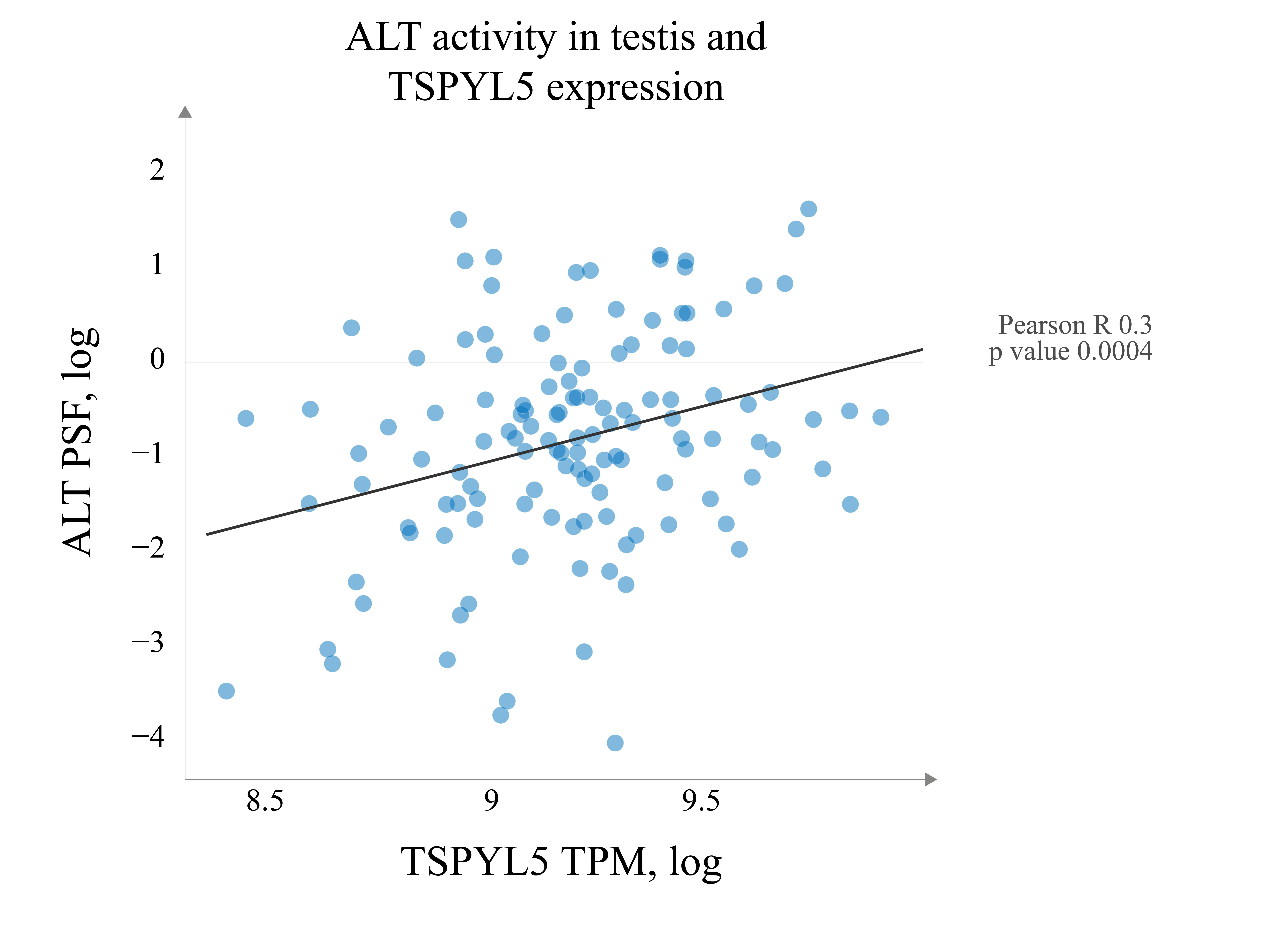


Figure S7. ALT pathway activity and expression of *TSPYL5*. ALT pathway PSF values show slight correlation (Pearson correlation R 0.03) with the expression of the testis-specific Y-encoded-like protein 5 (*TSPYL5*).

Table S4. References used for pathway curation, where relevance of a gene for the ALT pathway was measured by either the APB or the C-circle assay, or both.

| Both, APB and C-circle | Only APB | Only C-circle |
| --- | --- | --- |
| (Clynes et al., 2015)  (Conomos et al., 2014)  (Deeg et al., 2016)  (Dilley et al., 2016)  (Flynn et al., 2015)  (Jia-Min Zhang et al., 2019)  (Sobinoff et al., 2017) | (Cho et al., 2014)  (Chung et al., 2012)  (Jiang et al., 2005)  (Potts and Yu, 2007) | (Lovejoy et al., 2012)  (Min et al., 2019) |

# References

Aragón, L. (2018). The Smc5/6 Complex: New and Old Functions of the Enigmatic Long-Distance Relative. *Annu. Rev. Genet.* 52, 89–107. doi:10.1146/annurev-genet-120417-031353.

Boyraz, B., Moon, D. H., Segal, M., Muosieyiri, M. Z., Aykanat, A., Tai, A. K., et al. (2016). Posttranscriptional manipulation of TERC reverses molecular hallmarks of telomere disease. *J. Clin. Invest.* 126, 3377–82. doi:10.1172/JCI87547.

Cayuela, M. L., Flores, J. M., and Blasco, M. A. (2005). The telomerase RNA component Terc is required for the tumour-promoting effects of Tert overexpression. *EMBO Rep.* 6, 268–274. doi:10.1038/sj.embor.7400359.

Chen, L.-Y., Redon, S., and Lingner, J. (2012). The human CST complex is a terminator of telomerase activity. *Nature* 488, 540–4. doi:10.1038/nature11269.

Chen, L., Roake, C. M., Freund, A., Batista, P. J., Tian, S., Yin, Y. A., et al. (2018). An Activity Switch in Human Telomerase Based on RNA Conformation and Shaped by TCAB1. *Cell* 174, 218-230.e13. doi:10.1016/j.cell.2018.04.039.

Cho, N. W., Dilley, R. L., Lampson, M. A., and Greenberg, R. A. (2014). Interchromosomal homology searches drive directional ALT telomere movement and synapsis. *Cell* 159, 108–121. doi:10.1016/j.cell.2014.08.030.

Chung, I., Osterwald, S., Deeg, K. I., and Rippe, K. (2012). PML body meets telomere: the beginning of an ALTernate ending? *Nucleus* 3, 263–75. doi:10.4161/nucl.20326.

Clynes, D., Jelinska, C., Xella, B., Ayyub, H., Scott, C., Mitson, M., et al. (2015). Suppression of the alternative lengthening of telomere pathway by the chromatin remodelling factor ATRX. *Nat. Commun.* 6, 7538. doi:10.1038/ncomms8538.

Cohen, S. B., Graham, M. E., Lovrecz, G. O., Bache, N., Robinson, P. J., and Reddel, R. R. (2007). Protein composition of catalytically active human telomerase from immortal cells. *Science* 315, 1850–3. doi:10.1126/science.1138596.

Conomos, D., Reddel, R. R., and Pickett, H. A. (2014). NuRD-ZNF827 recruitment to telomeres creates a molecular scaffold for homologous recombination. doi:10.1038/nsmb.2877.

Deeg, K. I., Chung, I., Bauer, C., and Rippe, K. (2016). Cancer Cells with Alternative Lengthening of Telomeres Do Not Display a General Hypersensitivity to ATR Inhibition. *Front. Oncol.* 6, 186. doi:10.3389/fonc.2016.00186.

Dilley, R. L., Verma, P., Cho, N. W., Winters, H. D., Wondisford, A. R., and Greenberg, R. A. (2016). Break-induced telomere synthesis underlies alternative telomere maintenance. *Nature* 539, 54–58. doi:10.1038/nature20099.

Dimitrova, N., and de Lange, T. (2009). Cell Cycle-Dependent Role of MRN at Dysfunctional Telomeres: ATM Signaling-Dependent Induction of Nonhomologous End Joining (NHEJ) in G1 and Resection-Mediated Inhibition of NHEJ in G2. *Mol. Cell. Biol.* 29, 5552–5563. doi:10.1128/mcb.00476-09.

Dyer, M. A., Qadeer, Z. A., Valle-Garcia, D., and Bernstein, E. (2017). ATRX and DAXX: Mechanisms and Mutations. *Cold Spring Harb. Perspect. Med.* 7, a026567. doi:10.1101/cshperspect.a026567.

Flynn, R. L., Chang, S., and Zou, L. (2012). RPA and POT1: friends or foes at telomeres? *Cell Cycle* 11, 652–7. doi:10.4161/cc.11.4.19061.

Flynn, R. L., Cox, K. E., Jeitany, M., Wakimoto, H., Bryll, A. R., Ganem, N. J., et al. (2015). Alternative lengthening of telomeres renders cancer cells hypersensitive to ATR inhibitors. *Science* 347, 273–7. doi:10.1126/science.1257216.

Frohnert, C., Hutten, S., Wälde, S., Nath, A., and Kehlenbach, R. H. (2014). Importin 7 and Nup358 promote nuclear import of the protein component of human telomerase. *PLoS One* 9. doi:10.1371/journal.pone.0088887.

Jeong, S. A., Kim, K., Lee, J. H., Cha, J. S., Khadka, P., Cho, H. S., et al. (2015). Akt-mediated phosphorylation increases the binding affinity of hTERT for importin α to promote nuclear translocation. *J. Cell Sci.* doi:10.1242/jcs.166132.

Jia-Min Zhang, A., Yadav, T., Ouyang, J., Lan, L., and Zou Correspondence, L. (2019). Alternative Lengthening of Telomeres through Two Distinct Break-Induced Replication Pathways. *CellReports* 26, 955-968.e3. doi:10.1016/j.celrep.2018.12.102.

Jiang, W.-Q., Zhong, Z.-H., Henson, J. D., Neumann, A. A., Chang, A. C.-M., and Reddel, R. R. (2005). Suppression of Alternative Lengthening of Telomeres by Sp100-Mediated Sequestration of the MRE11/RAD50/NBS1 Complex. *Mol. Cell. Biol.* 25, 2708–2721. doi:10.1128/MCB.25.7.2708-2721.2005.

Lafrance-Vanasse, J., Williams, G. J., and Tainer, J. A. (2015). Envisioning the dynamics and flexibility of Mre11-Rad50-Nbs1 complex to decipher its roles in DNA replication and repair. *Prog. Biophys. Mol. Biol.* 117, 182–193. doi:10.1016/j.pbiomolbio.2014.12.004.

Lee, J. H., Khadka, P., Baek, S. H., and Chung, I. K. (2010). CHIP promotes hTERT degradation and negatively regulates telomerase activity. *J. Biol. Chem.* 285, 42033–42045. doi:10.1074/jbc.M110.149831.

Lovejoy, C. A., Li, W., Reisenweber, S., Thongthip, S., Bruno, J., de Lange, T., et al. (2012). Loss of ATRX, genome instability, and an altered DNA damage response are hallmarks of the alternative lengthening of telomeres pathway. *PLoS Genet.* 8, e1002772. doi:10.1371/journal.pgen.1002772.

Min, J., Wright, W. E., and Shay, J. W. (2019). Clustered telomeres in phase-separated nuclear condensates engage mitotic DNA synthesis through BLM and RAD52. *Genes Dev.* 33, 814–827. doi:10.1101/gad.324905.119.

Moon, D. H., Segal, M., Boyraz, B., Guinan, E., Hofmann, I., Cahan, P., et al. (2015). Poly(A)-specific ribonuclease (PARN) mediates 3′-end maturation of the telomerase RNA component. *Nat. Genet.* 47, 1482–1488. doi:10.1038/ng.3423.

Potts, P. R., and Yu, H. (2007). The SMC5/6 complex maintains telomere length in ALT cancer cells through SUMOylation of telomere-binding proteins. *Nat. Struct. Mol. Biol.* 14, 581–90. doi:10.1038/nsmb1259.

Schmidt, J. C., and Cech, T. R. (2015). Human telomerase: Biogenesis, trafficking, recruitment, and activation. *Genes Dev.* 29, 1095–1105. doi:10.1101/gad.263863.115.

Sobinoff, A. P., Allen, J. A., Neumann, A. A., Yang, S. F., Walsh, M. E., Henson, J. D., et al. (2017). BLM and SLX4 play opposing roles in recombination‐dependent replication at human telomeres. *EMBO J.* 36, 2907–2919. doi:10.15252/embj.201796889.

Tseng, C.-K., Wang, H.-F., Burns, A. M., Schroeder, M. R., Gaspari, M., and Baumann, P. (2015). Human Telomerase RNA Processing and Quality Control. *Cell Rep.* 13, 2232–2243. doi:10.1016/j.celrep.2015.10.075.

Venteicher, A. S., Meng, Z., Mason, P. J., Veenstra, T. D., and Artandi, S. E. (2008). Identification of ATPases Pontin and Reptin as Telomerase Components Essential for Holoenzyme Assembly. *Cell* 132, 945–957. doi:10.1016/j.cell.2008.01.019.

Zhu, X. D., Niedernhofer, L., Kuster, B., Mann, M., Hoeijmakers, J. H. J., and De Lange, T. (2003). ERCC1/XPF Removes the 3′ Overhang from Uncapped Telomeres and Represses Formation of Telomeric DNA-Containing Double Minute Chromosomes. *Mol. Cell*. doi:10.1016/S1097-2765(03)00478-7.
