# Supplementary figures and images for: Telomere Maintenance Pathway Activity Analysis Enables Tissue- and Gene-Level Inferences
Source: Front Genet. 2021 Apr 7;12:662464. doi: 10.3389/fgene.2021.662464 (PMC8058386; doi:10.3389/fgene.2021.662464)

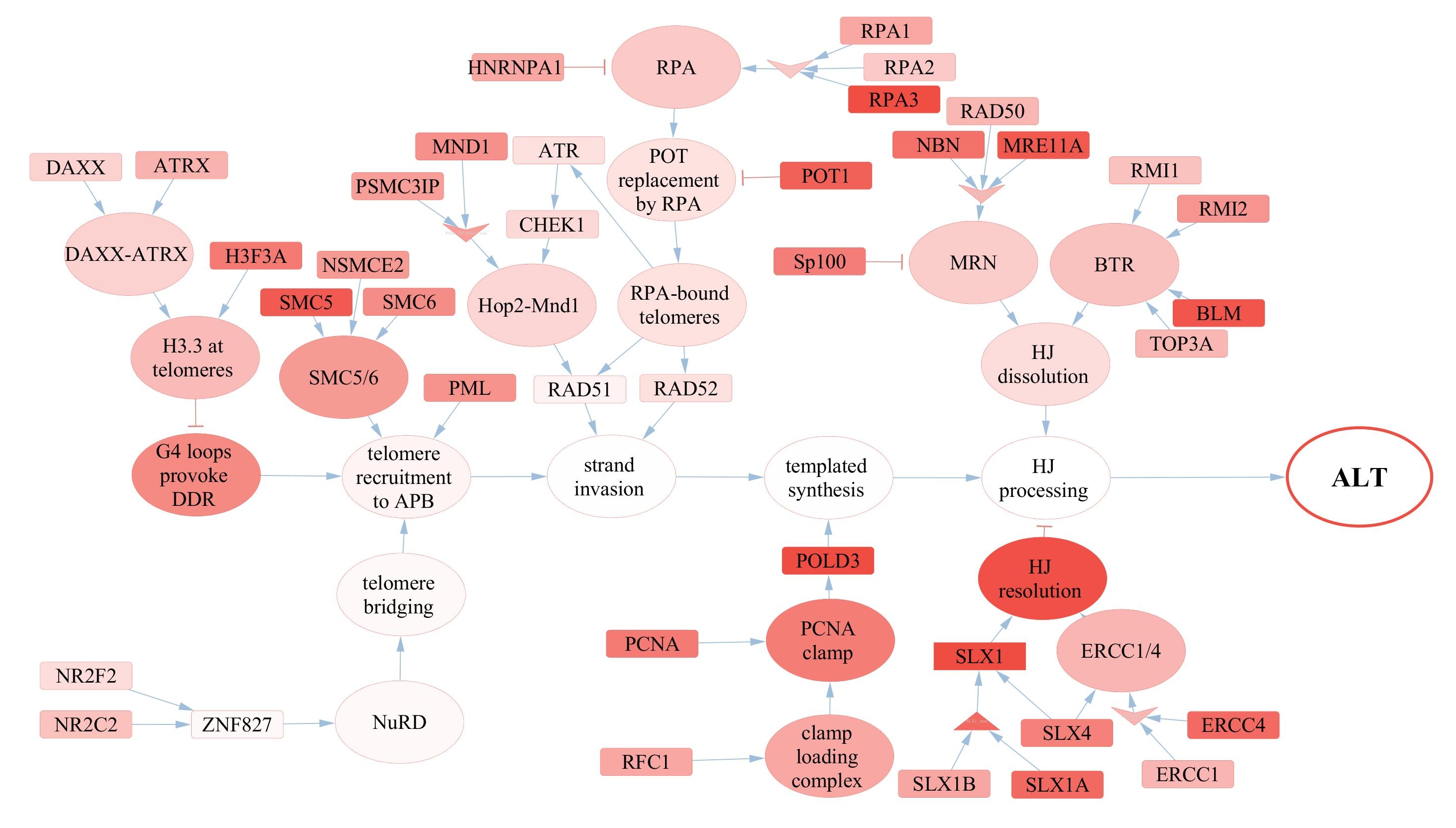

Supplement: Supplementary file 3 [file Data_Sheet_3.ZIP › Supplementary data 3/cell_lines/ALT_5637_1.jpg]

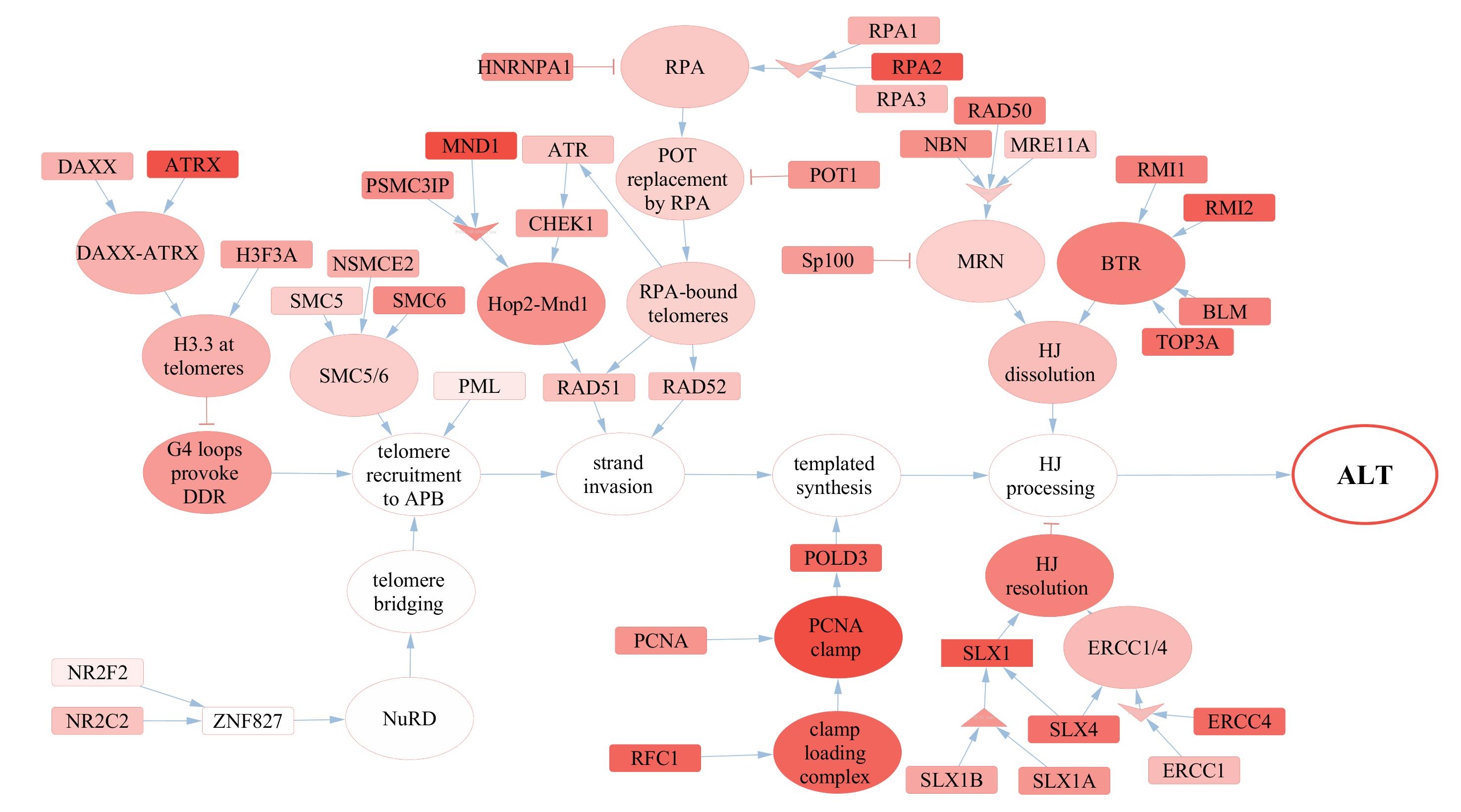

Supplement: Supplementary file 3 [file Data_Sheet_3.ZIP › Supplementary data 3/cell_lines/ALT_C33_1.jpg]

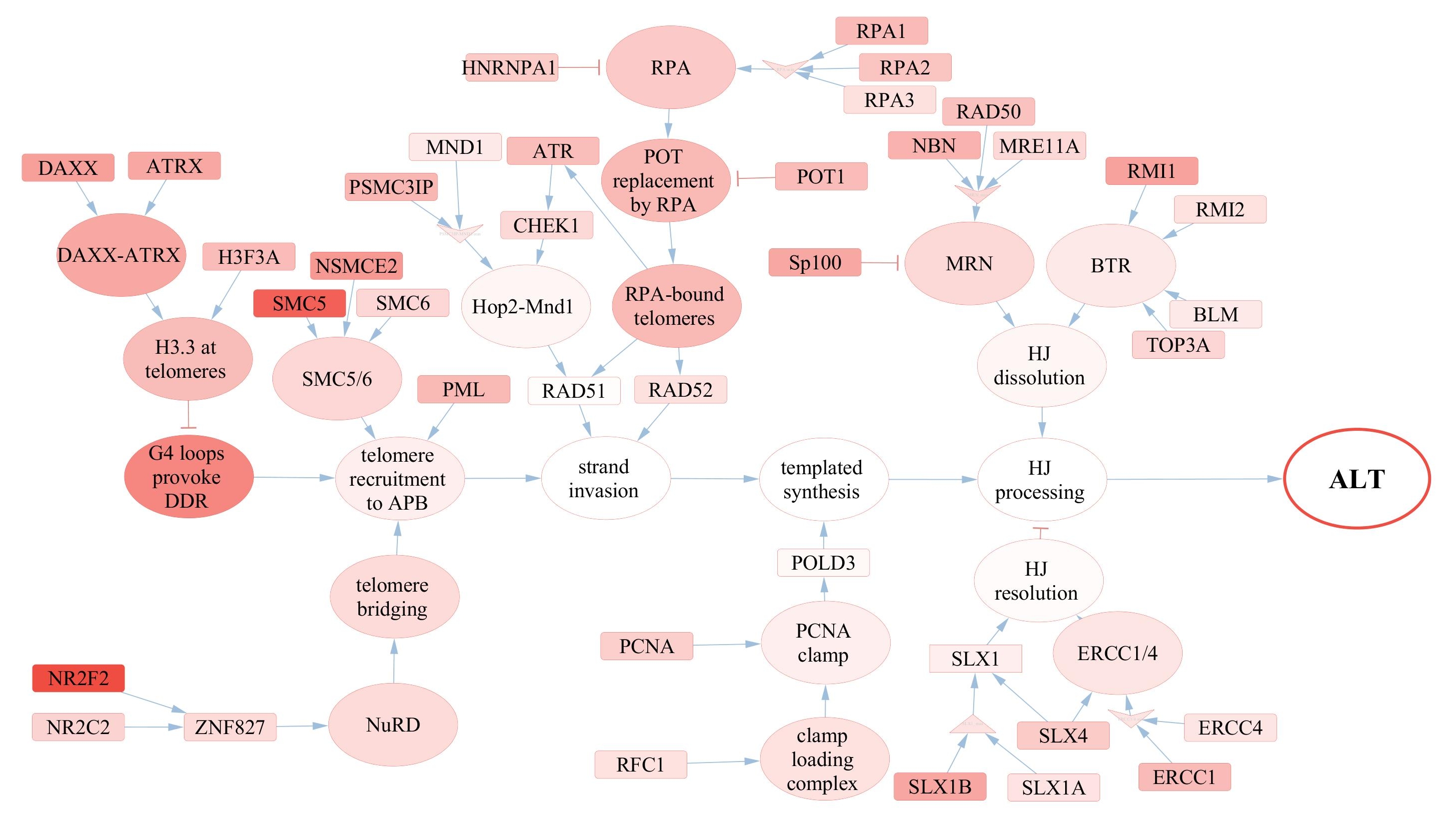

Supplement: Supplementary file 3 [file Data_Sheet_3.ZIP › Supplementary data 3/cell_lines/ALT_IMR90_1.jpg]

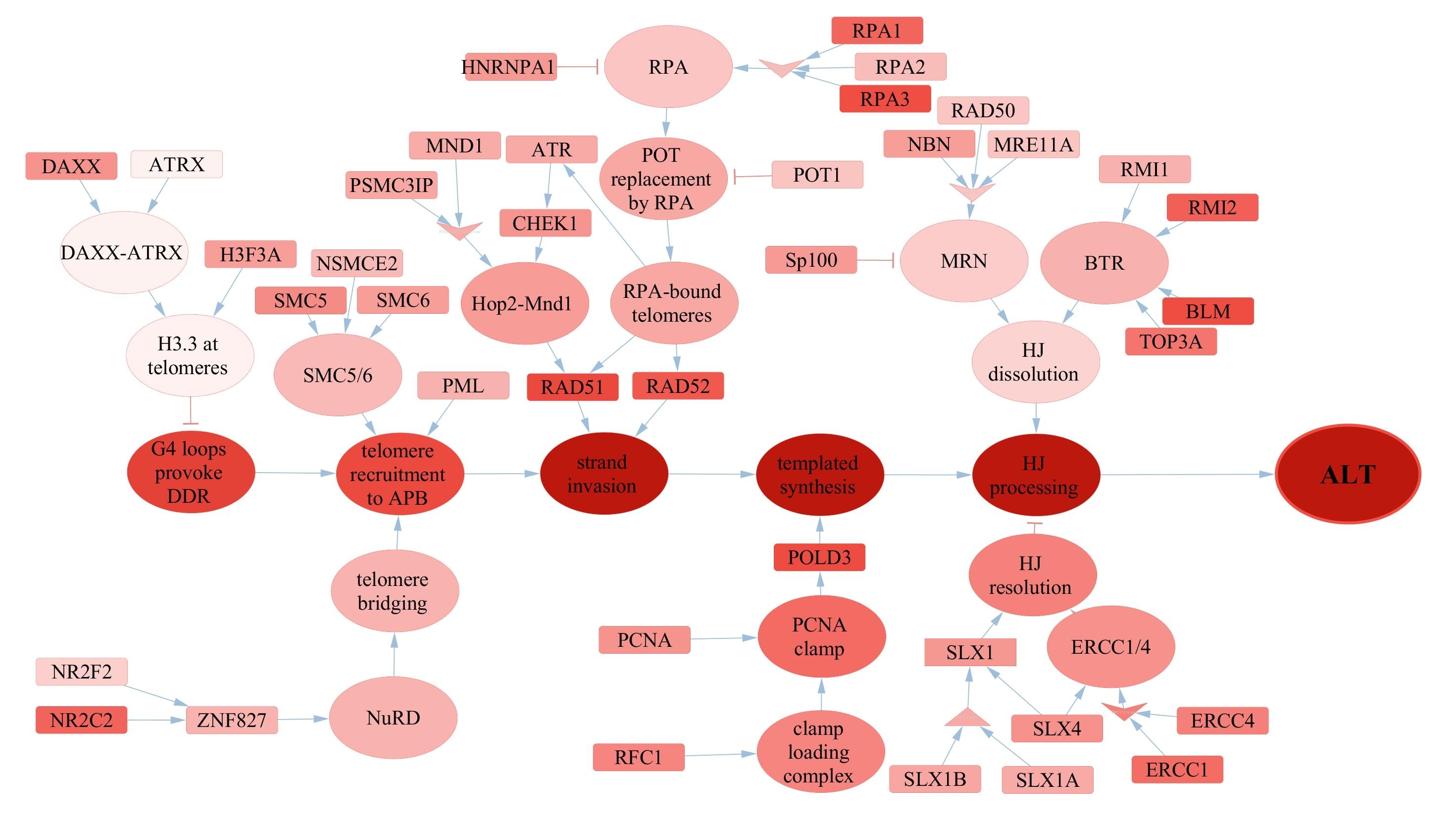

Supplement: Supplementary file 3 [file Data_Sheet_3.ZIP › Supplementary data 3/cell_lines/ALT_SUSM1_1.jpg]

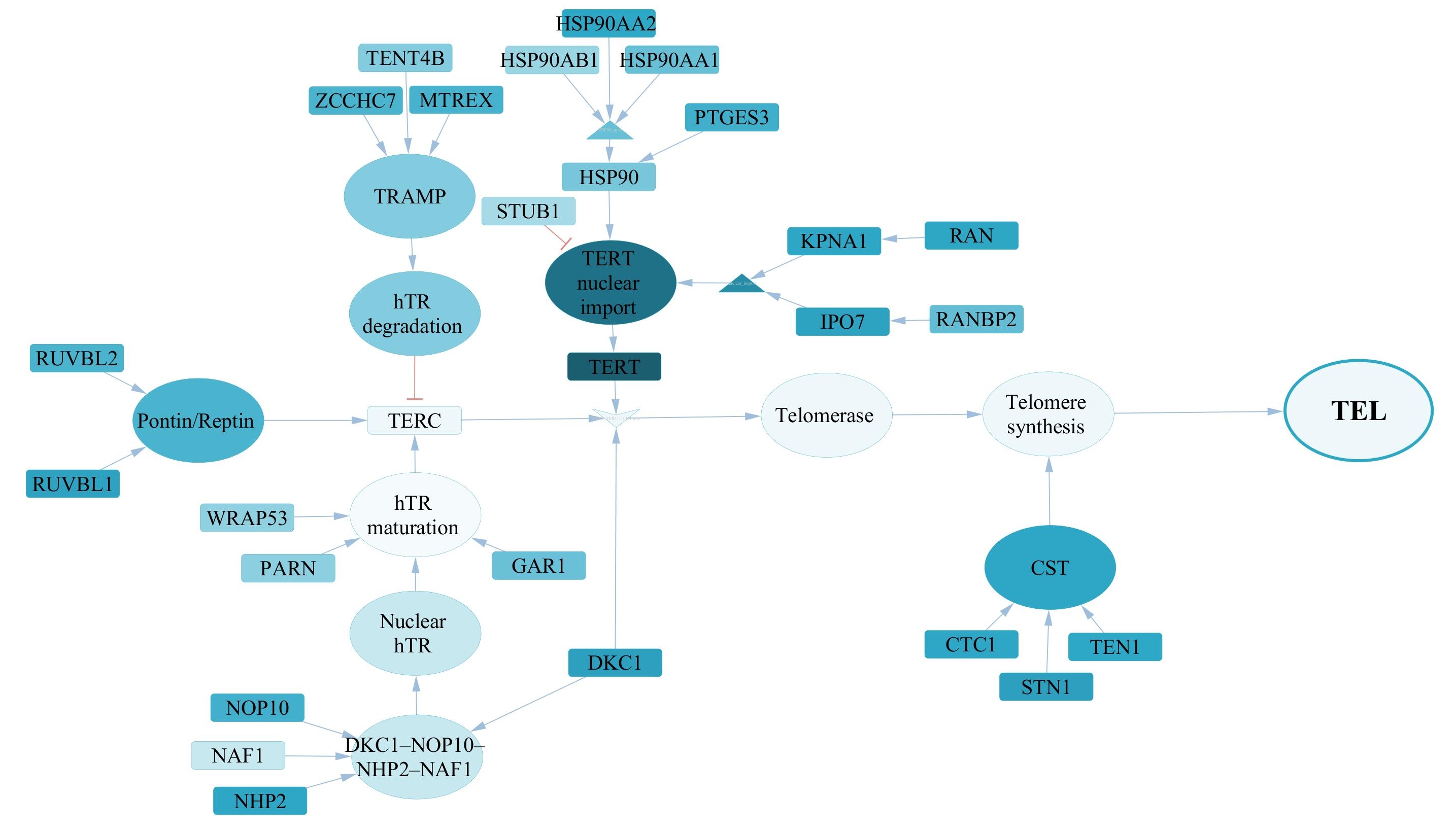

Supplement: Supplementary file 3 [file Data_Sheet_3.ZIP › Supplementary data 3/cell_lines/TEL_5637_1.jpg]

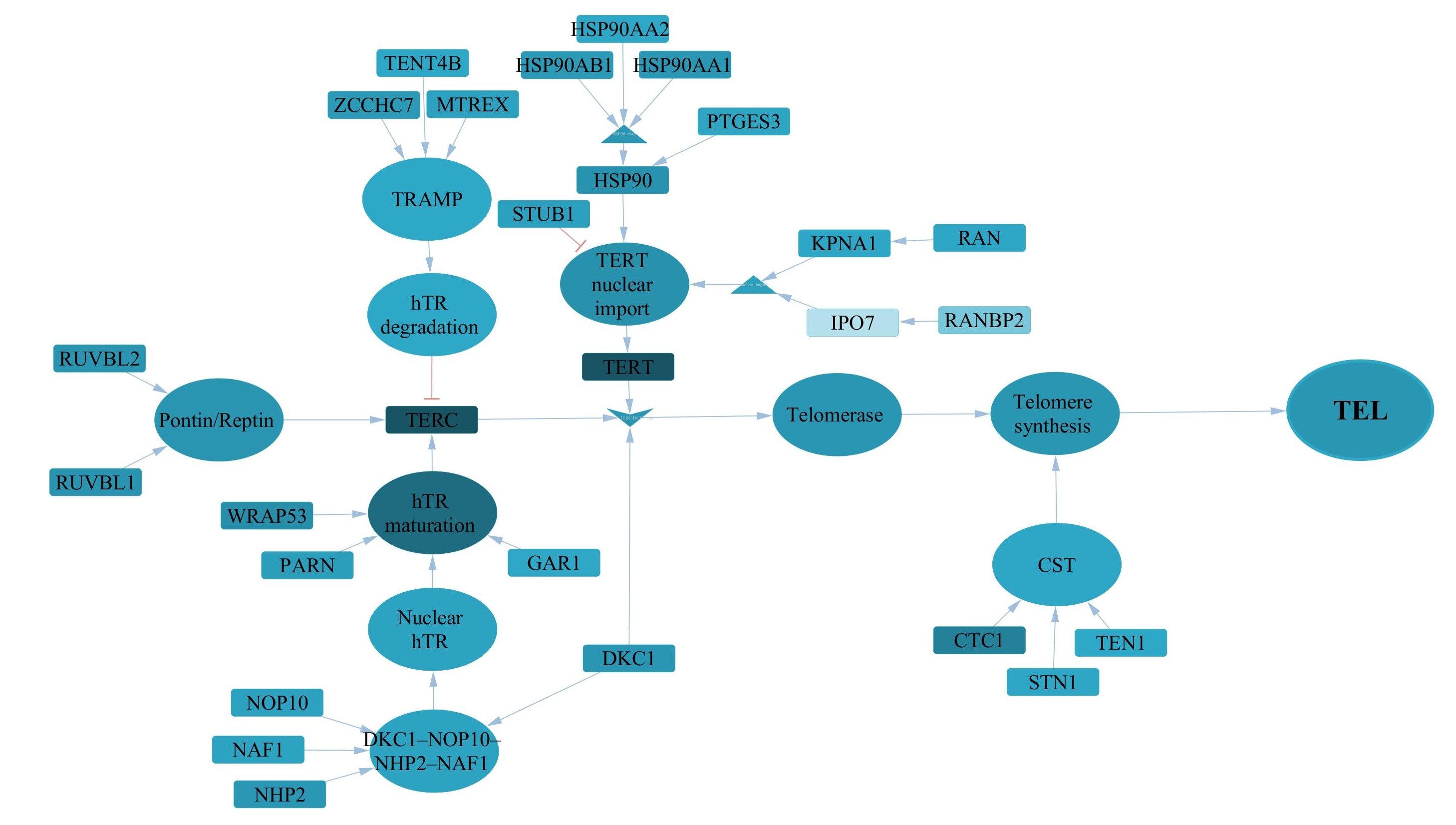

Supplement: Supplementary file 3 [file Data_Sheet_3.ZIP › Supplementary data 3/cell_lines/TEL_C33_1.jpg]

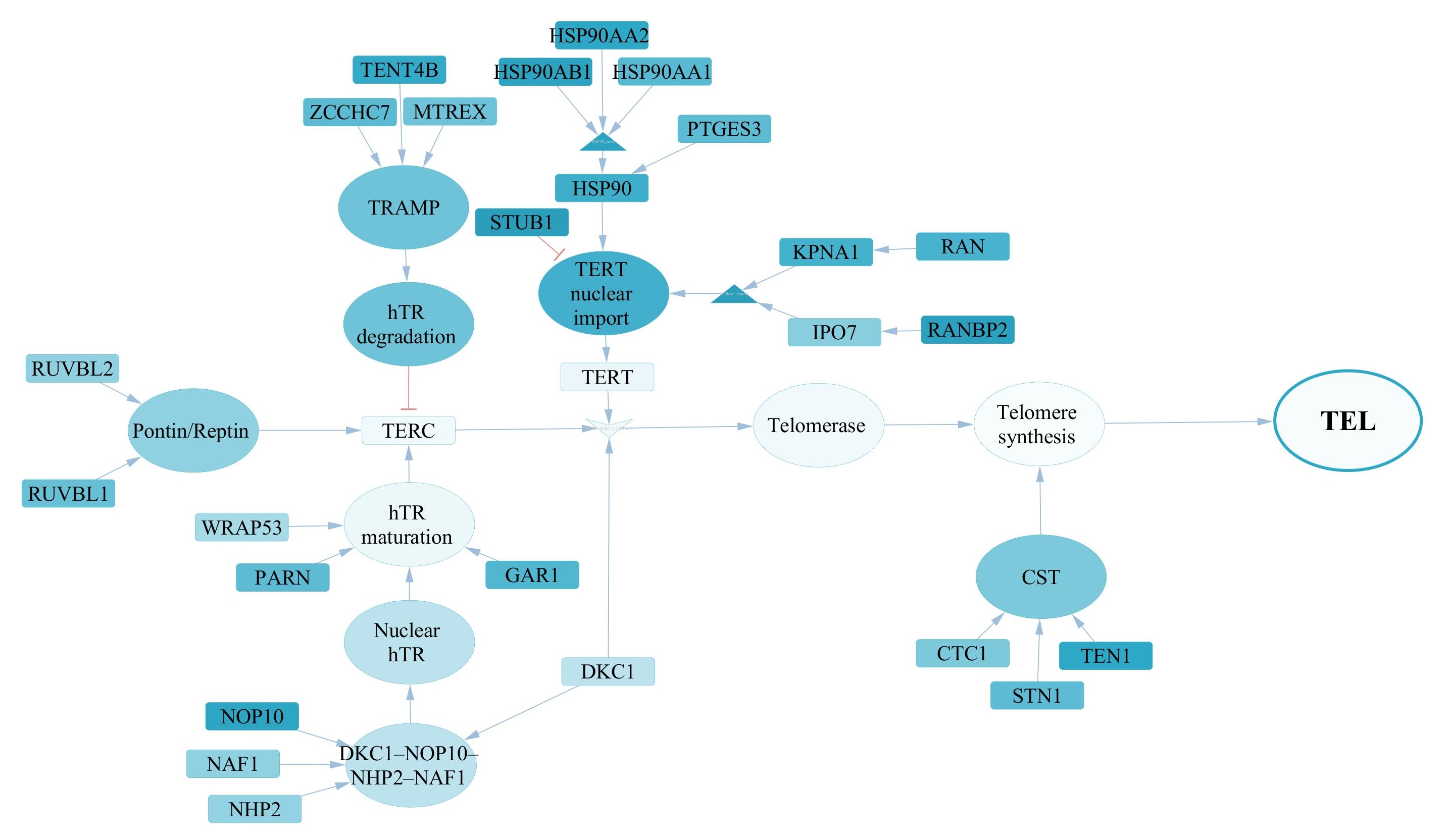

Supplement: Supplementary file 3 [file Data_Sheet_3.ZIP › Supplementary data 3/cell_lines/TEL_IMR90_1.jpg]

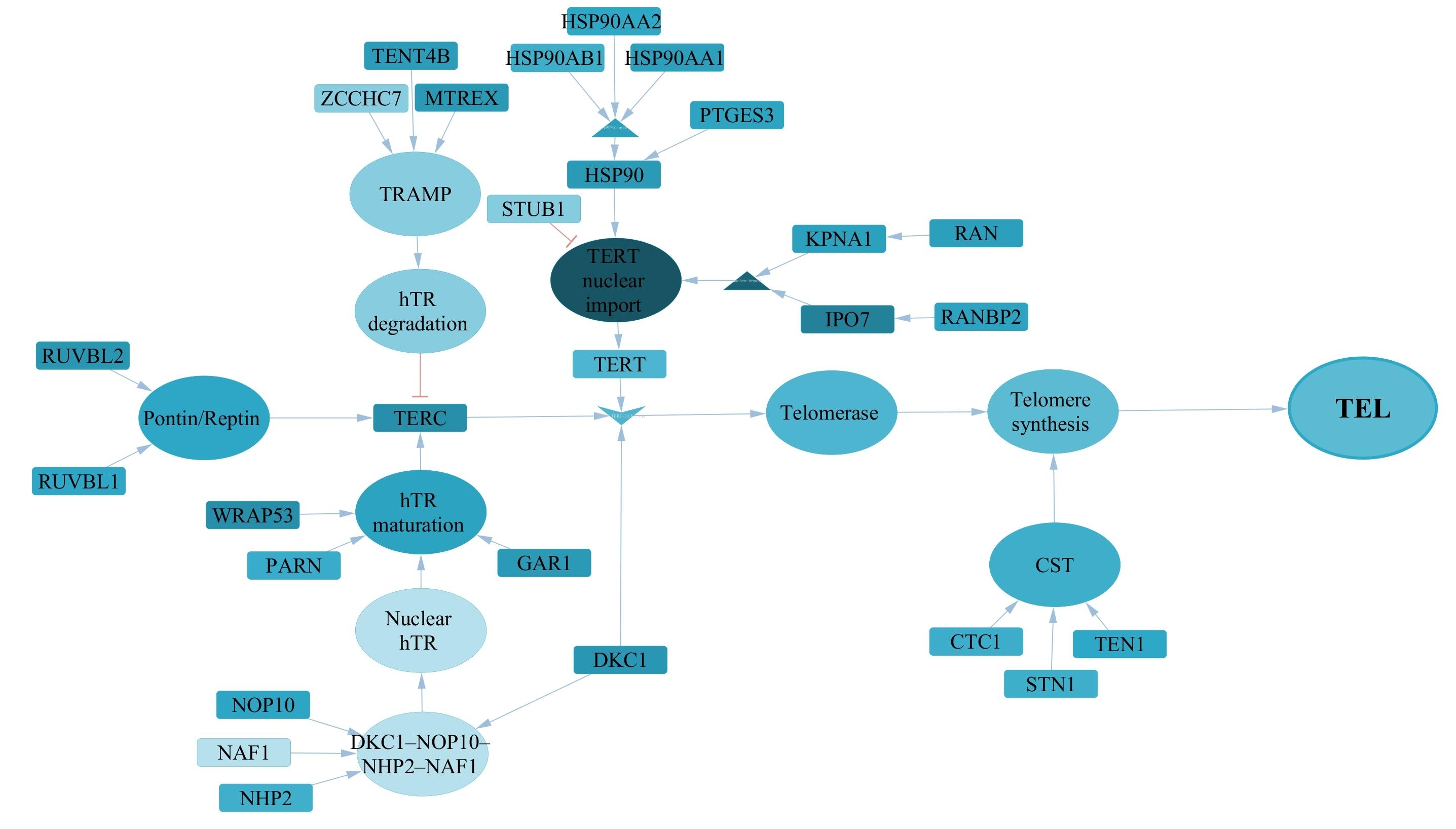

Supplement: Supplementary file 3 [file Data_Sheet_3.ZIP › Supplementary data 3/cell_lines/TEL_SUSM1_1.jpg]

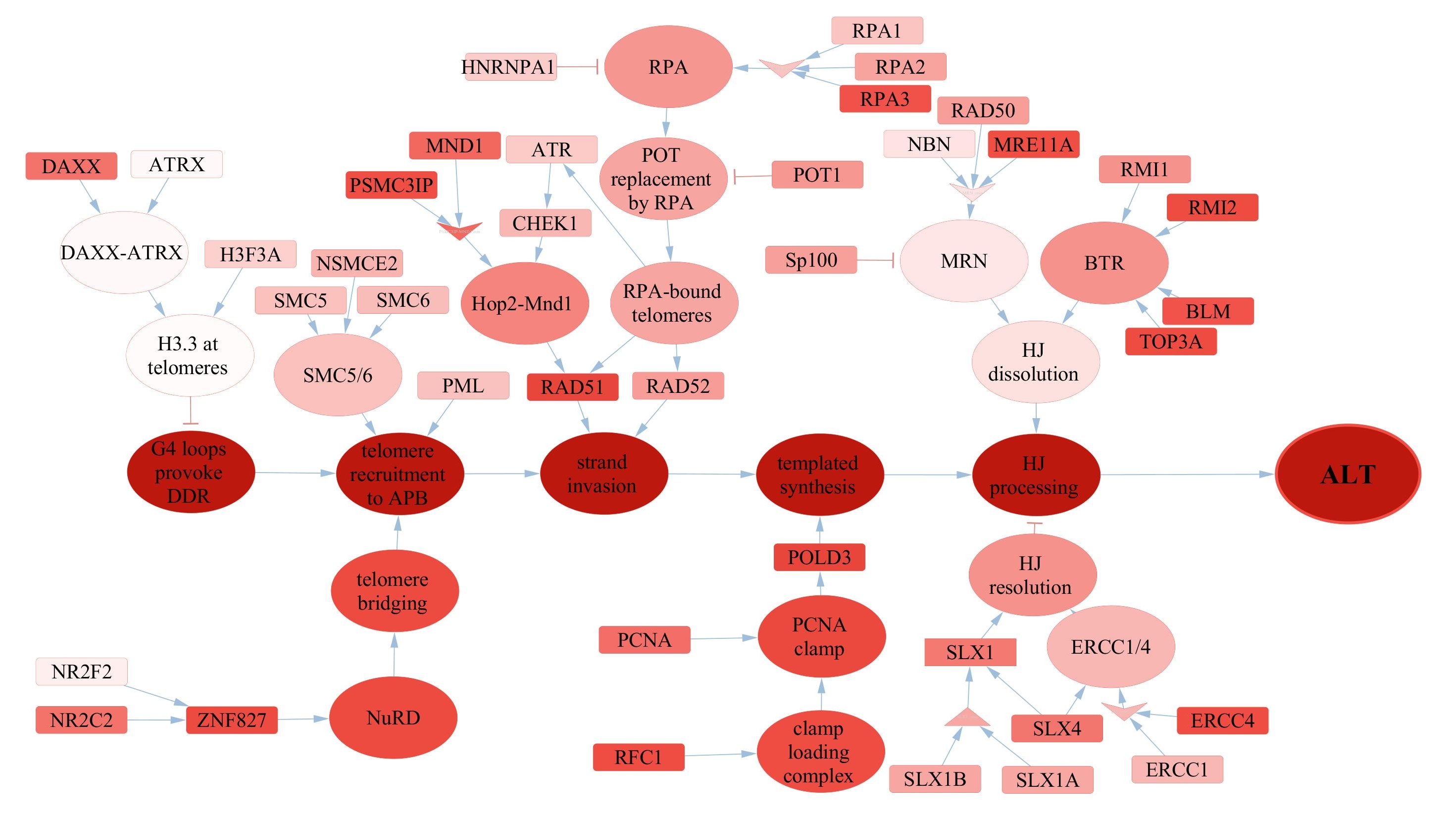

Supplement: Supplementary file 3 [file Data_Sheet_3.ZIP › Supplementary data 3/liposarcoma/ALT_A1.jpg]

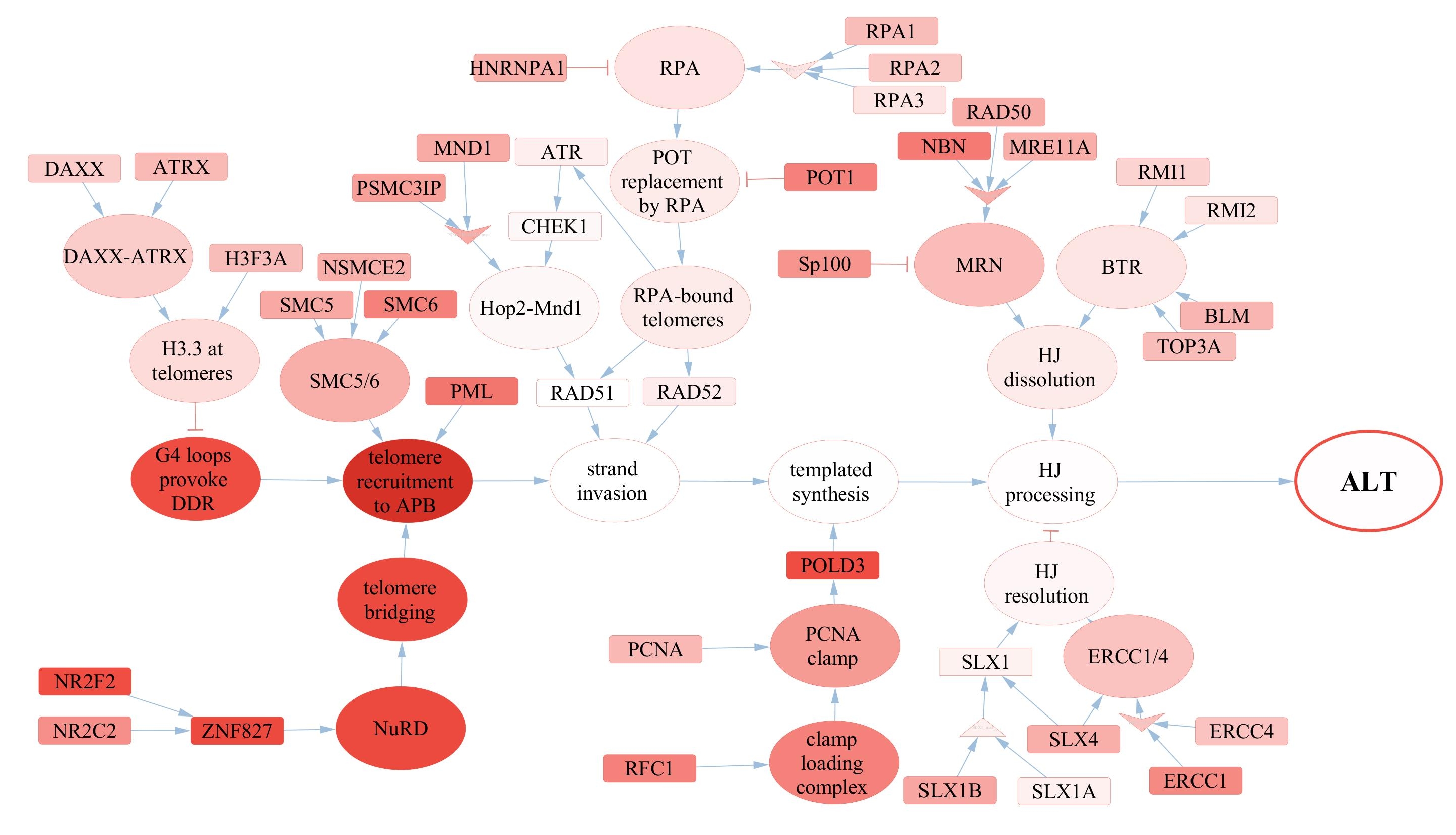

Supplement: Supplementary file 3 [file Data_Sheet_3.ZIP › Supplementary data 3/liposarcoma/ALT_A7.jpg]

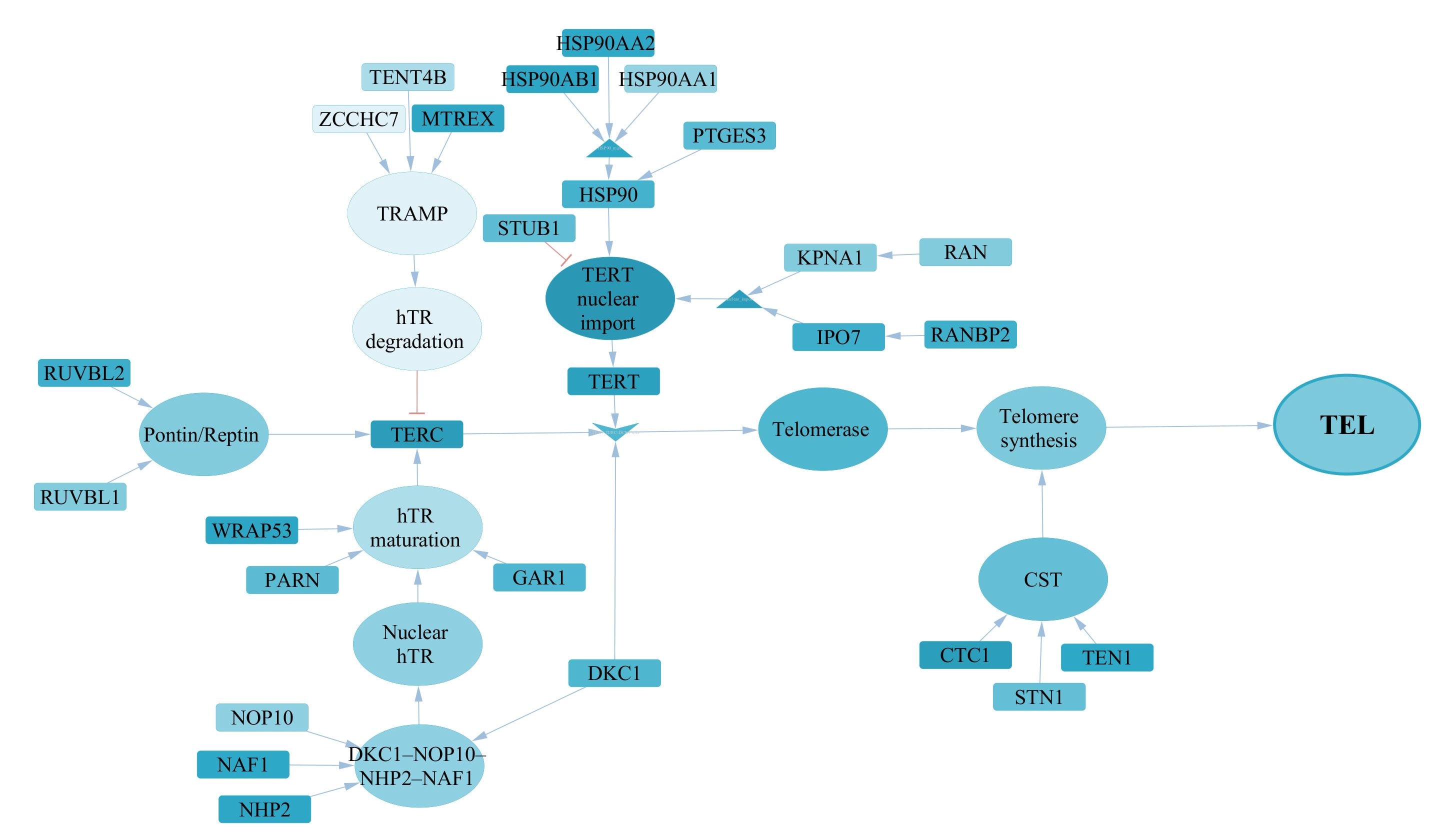

Supplement: Supplementary file 3 [file Data_Sheet_3.ZIP › Supplementary data 3/liposarcoma/ALT_T5.jpg]

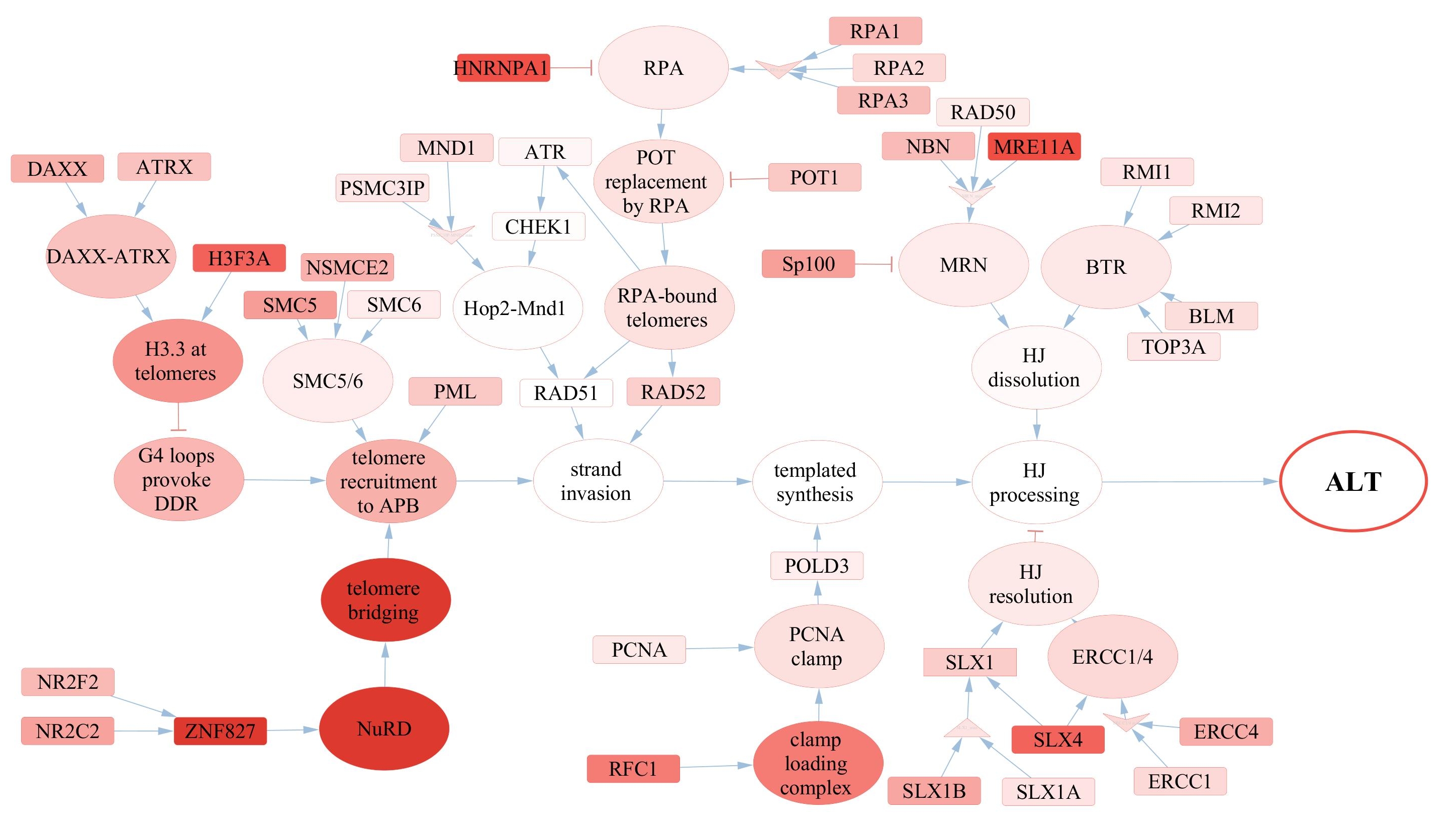

Supplement: Supplementary file 3 [file Data_Sheet_3.ZIP › Supplementary data 3/liposarcoma/ALT_T8.jpg]

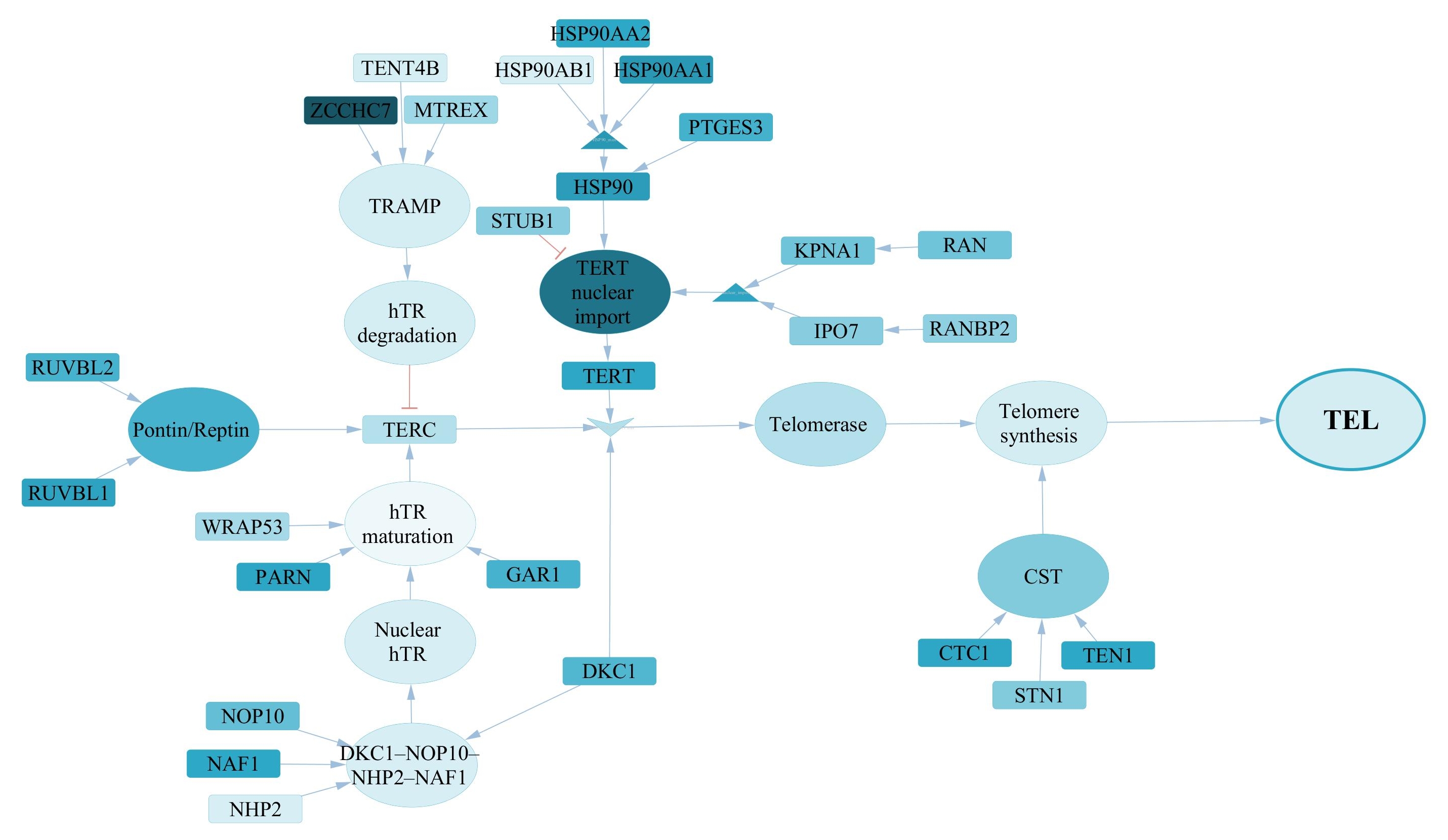

Supplement: Supplementary file 3 [file Data_Sheet_3.ZIP › Supplementary data 3/liposarcoma/TEL_A1.jpg]

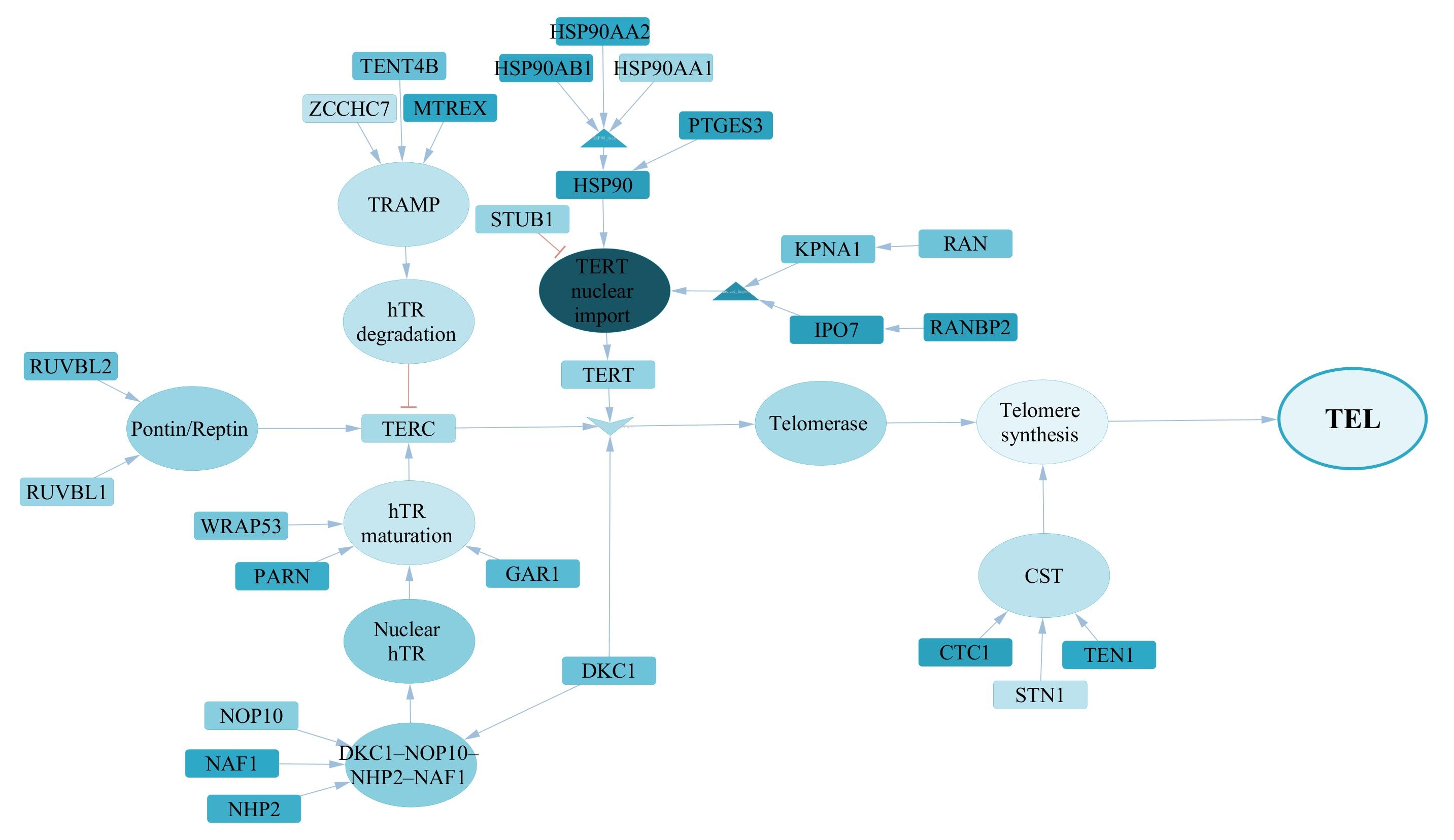

Supplement: Supplementary file 3 [file Data_Sheet_3.ZIP › Supplementary data 3/liposarcoma/TEL_A7.jpg]

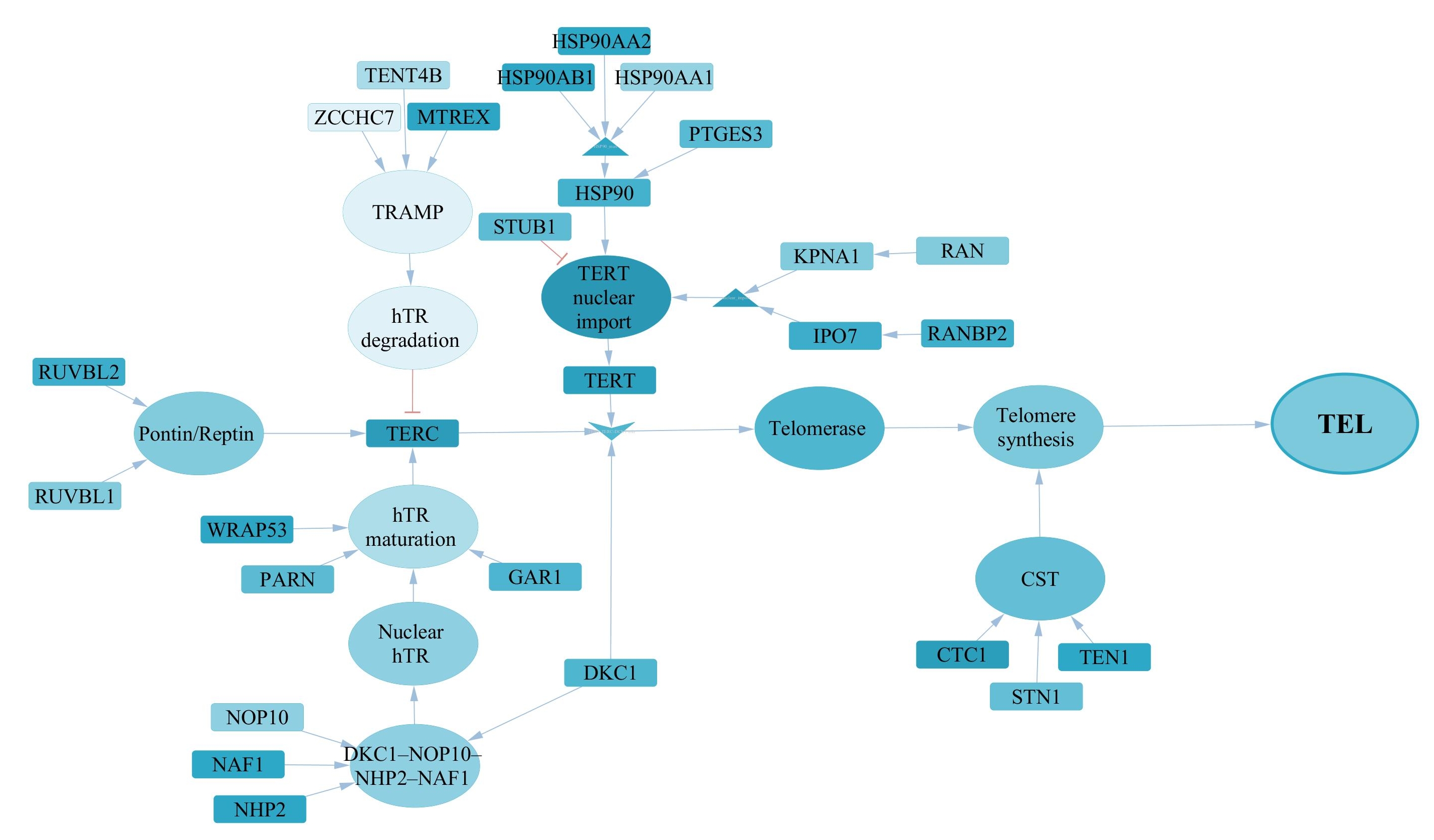

Supplement: Supplementary file 3 [file Data_Sheet_3.ZIP › Supplementary data 3/liposarcoma/TEL_T5.jpg]

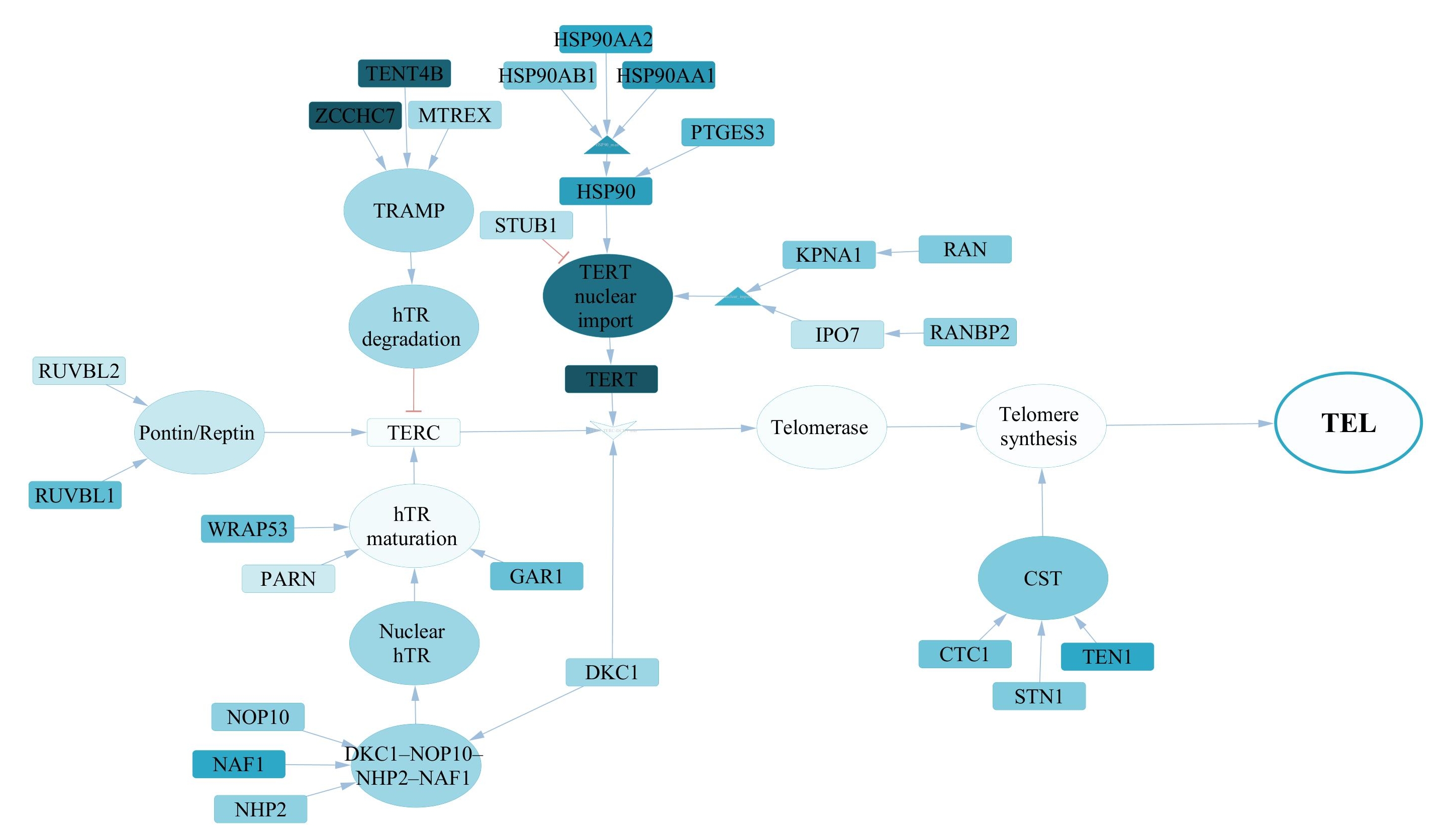

Supplement: Supplementary file 3 [file Data_Sheet_3.ZIP › Supplementary data 3/liposarcoma/TEL_T8.jpg]
